# Accelerating Cleavage Activity of CRISPR-Cas13 System on a Microfluidic Chip for Rapid Detection of RNA

**DOI:** 10.1021/acs.analchem.5c00256

**Published:** 2025-04-30

**Authors:** Jongmin Kim, Ajymurat Orozaliev, Sarah Sahloul, Anh-Duc Van, Van-Truong Dang, Van-Sang Pham, Yujeong Oh, Ibrahim Chehade, Mohamed Al-Sayegh, Yong-Ak Song

**Affiliations:** † Division of Engineering, New York University Abu Dhabi, P.O. Box 129188, Abu Dhabi, UAE; ‡ Department of Mechanical and Aerospace Engineering, New York University Tandon School of Engineering, New York, New York 11201, United States; § School of Mechanical Engineering, Hanoi University of Science and Technology, No. 1 Daicoviet Road, Hanoi 100000, Vietnam; ∥ Division of Science, New York University Abu Dhabi, P.O. Box 129188, Abu Dhabi, UAE; ⊥ Department of Chemical and Biomolecular Engineering, New York University Tandon School of Engineering, Brooklyn, New York 11201, United States; # Department of Biomedical Engineering, New York University Tandon School of Engineering, Brooklyn, New York 11201, United States

## Abstract

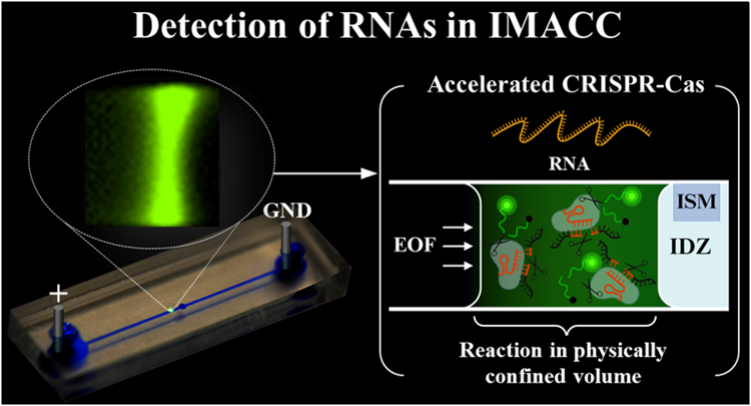

It is extremely advantageous to detect nucleic acid levels in the early phases of disease management; such early detection facilitates timely treatment, and it can prevent altogether certain cancers and infectious diseases. A simple, rapid, and versatile detection platform without enzymatic amplification for both short and long sequences would be highly desirable in this regard. Our study addresses this need by introducing IMACC, an ICP-based Microfluidic Accelerator Combined with CRISPR, for amplification-free nucleic acid detection. It exploits electrokinetically induced ion concentration polarization (ICP) to concentrate target nucleic acids and CRISPR reagents near the depletion zone boundary within a microfluidic channel. This localized accumulation accelerates the CRISPR-guided promiscuous cleavage of reporter molecules while enhancing their fluorescence signals simultaneously. Simultaneous accumulation of RNA and ribonucleoproteins (RNP) in confined spaces was validated experimentally and numerically, showing overlapping regions. IMACC enabled detection of miRNA-21 (22 bp) down to 10 pM within 2 min of ICP. IMACC ensured CRISPR specificity (single mismatch (*N* = 1) sensitivity) during ICP, as shown by off-target and mismatch sequence experiments. IMACC was applied to long RNA samples (i.e., SARS-CoV-2), but it statistically remained challenging at this point due to nonlinear intensity trends with copy numbers and large deviations. IMACC enabled rapid detection of short RNAs such as microRNAs using only basic CRISPR reagents in a single microfluidic channel, eliminating the need for extra enzymes or buffer sets, streamlining workflow and reducing turnaround time. IMACC has the potential to advance CRISPR diagnostics and holds promise for improved detection and future prescreening applications.

Fast and accurate nucleic acid detection is critical for early disease diagnosis, enabling effective treatment of severe illnesses like cancer and preventing infectious disease spread.^1^ The COVID-19 pandemic underscored the need for rapid and precise nucleic acid testing for effective disease control.^2^ However, disease-related nucleic acid biomarkers (DNA or RNA) are typically present in very low quantities, especially in early stages, requiring a prior amplification for detection. Polymerase Chain Reaction (PCR) remains the gold standard due to its high accuracy, but it is slow, expensive, and requires skilled personnel and specialized equipment.^3^ Isothermal methods like Loop-Mediated Isothermal Amplification (LAMP) and Recombinase Polymerase Amplification (RPA) reduce equipment needs but suffer from lower specificity, requiring additional processing steps.^4^ Next-generation sequencing (NGS) allows large-scale analysis but is costly and time-intensive, limiting its feasibility for early disease diagnosis.^5^ For the recent years, clustered regularly interspaced short palindromic repeat (CRISPR) based diagnostics have emerged as a powerful alternative, offering greater flexibility and single-base-pair precision for DNA/RNA detection using CRISPR-Cas12 and Cas13 ribonucleoproteins (RNPs).^6^ DETECTR (Cas12a) and SHERLOCK (Cas13a) demonstrated excellent sensitivity for detecting SARS-CoV-2 RNA, about 20 aM (∼10 copies/μL) and about 2 aM (∼1 copy/μL), respectively.^7,8^ Although their limits of detection (LOD) match quantitative PCR (qPCR),^9^ they still require target amplification, increasing complexity and assay time. Efforts to develop direct CRISPR-based detection without amplification have so far been limited to picomolar sensitivity,^10^ which is inadequate for early disease diagnosis where femtomolar to attomolar detection is needed, particularly in small sample volumes (∼10 μL).^11^ To address this, innovative CRISPR approaches have been developed to boost sensitivity without amplification using chemical and physical methods. Chemically, multiple crRNAs^12^ and tandem nucleases (FIND-IT)^13^ improve detection but prolong assay times at low concentrations, which increase total turnaround time that likely limits rapid screening of disease at early stage. Physically, microfluidic confinement methods like SATORI (microwells)^14^ and picoliter droplets^15^ enhance sensitivity. However, each comes with certain constraints: SATORI requires multiple crRNAs, but sensitivity is still quite less (femtomolar range), while picoliter droplet assays rely on additional accessories (syringes, tubing, and surfactants), increasing potential cross-contamination risks. Moreover, picoliter droplets are end point assays requiring ∼1 h to reach the plateau phase, likely delaying turnaround time.

Microfluidics combined with electrokinetic phenomena, such as Isotachophoresis (ITP)^16^ and Ion Concentration Polarization (ICP)^17,18^ offers a promising confinement environment based on the balance of physical forces, leading to locally concentrating biomolecules, which can eliminate key drawbacks of enzymatic amplification, including stringent reagents, multistep processing, and false amplicon results. ITP-Cas12a^19^ enabled SARS-CoV-2 detection at 10 copies/μL but required RT-LAMP amplification and specific buffer combinations (Leading Electrolyte (LE) and Terminating Electrolyte (TE)). Meanwhile, ICP-dCas9 facilitated amplification-free detection of short DNA targets such as CCR5 (<200 bp)^20^ and oncogenic mutation of EGFR DNA^21^ but required fluorescence tagging. Instead, a simple platform without any complex buffer system or sample tagging would be highly desirable for the accelerated CRISPR detection of DNA or RNA at high sensitivity (femtomolecular to aM levels) in an amplification-free manner.

In this work, we demonstrate an ICP-based Microfluidic Accelerator Combined with CRISPR (IMACC) for the rapid and versatile CRISPR-Cas detection of nucleic acids in an amplification-free manner. Upon generating ICP by an electric field, target nucleic acids and CRISPR reagents, including Cas-crRNA complex (RNP) and reporter, are locally accumulated adjacent to the boundary of the ion depletion zone. This spatial confinement allows acceleration of CRISPR-guided reporter cleavage by reducing the diffusion length, as well as enhanced fluorescence signal resulting from the preconcentration of cleaved reporter molecules. Fluorescence analysis and numerical simulations confirmed that CRISPR components (i.e., RNA and RNP) accumulate and partially overlap during ICP in IMACC. This spatial confinement is likely to shorten diffusion lengths, enhance cleavage activity, and simultaneously accumulate cleaved dye, contributing to the improved reporter signal. A combination of these effects leads to an overall enhancement of detection sensitivity and speed. This favorable environment in IMACC allowed effective detection of short RNA (miRNA-21, 22 bp) down to 10 pM (∼6 × 10^6^ copies/μL) within 2 min of ICP. Furthermore, IMACC effectively maintained CRISPR specificity during ICP, ensuring minimal false signal generation from off-targets or mismatched sequences as small as *N* = 1. Next, IMACC was applied to assess detection feasibility for a long RNA (SARS-CoV-2, ∼30 kb), but results from both synthetic and infected patient samples revealed a nonlinear relationship between signal and copy numbers, with large deviations, resulting in no statistically significant difference between the signal and the noise. At the current state, therefore, IMACC is only a qualitative screening platform for short RNAs. In contrast to earlier methods requiring complex reagents or confinement strategies, IMACC operates with minimal components, such as a single type of crRNA and Cas enzyme and a standard buffer, in a single-channel microfluidic device. This simplicity makes IMACC a promising tool for rapid and efficient CRISPR-based nucleic acid detection.

## Experimental Section

### Preparation of Test Samples

Synthetic samples: miRNA-21 (10–10^5^ pM) and SARS-CoV-2 RNA (10^–1^–10^5^ copies/μL) were prepared via 1:10 serial dilution in nuclease-free water. CRISPR ribonucleoprotein (RNP) complexes were formed by incubating 0.5 μM LbuCas13a and 0.5 μM crRNA for each RNA target at room temperature for 10 min. The final master mix was prepared by combining with 10× reaction buffer, nuclease-free water, 4 μM poly U reporter, and 40 U/μL RNase inhibitor. Test sample solutions were prepared by mixing 2 μL of the RNA target with 8 μL of the master mix immediately before loading into the microfluidic accelerator. All sequences and reagent concentrations (stocks and final) are detailed in Tables S1 and S2.

### Preparation of Clinical Samples

Fourteen clinical samples, consisting of 7 healthy samples and 7 SARS-CoV-2-infected patient samples previously confirmed by RT-qPCR, were used to validate the feasibility of IMACC for clinical applications.^22^ Briefly, automatic RNA extractions were performed from nasopharyngeal (NP) swab samples using a Chemagic 360 automated nucleic acid extraction system (2024-0020, PerkinElmer, Waltham, MA) and the Chemagic Viral DNA/RNA 300 Kit H96 (CMG-1033S, PerkinElmer, Waltham). For all 14 of these extracted clinical samples, triplicate tests were performed using IMACC.

### IMACC: Fabrication, System Setup, and Detection Analysis

The device for IMACC was fabricated via conventional soft lithography and UV-induced photopolymerization. The detailed fabrication procedure can be found in Figure S1. Before loading samples, the device was plasma-treated at 800 mTorr for 1 min and bonded to a glass substrate. 10 μL of test sample solution was evenly split into each reservoir. The accelerator was preheated at 37 °C for 10 min using an ITO heater (HI-57Dp and mTCII, Cell MicroControls, Norfolk, VA). Then, ICP was initiated by applying 60 V (*E* = 45.5 V/cm) using a Keithley source meter (2400 SMU, Tektronix, Beaverton, OR) through two platinum wire electrodes (0.508 mm, Beantown Chemical, Hudson, NH), precisely placed in each reservoir to ensure a consistent electric field. The fluorescence signal during ICP was monitored using a Nikon inverted epi-fluorescence microscope, and the signals were analyzed by Nikon software and NIH ImageJ, as detailed in Supporting Information.

## Results and Discussion

### Operation of ICP-Based Microfluidic Accelerator Combined with CRISPR (IMACC)

**Figure 1:**
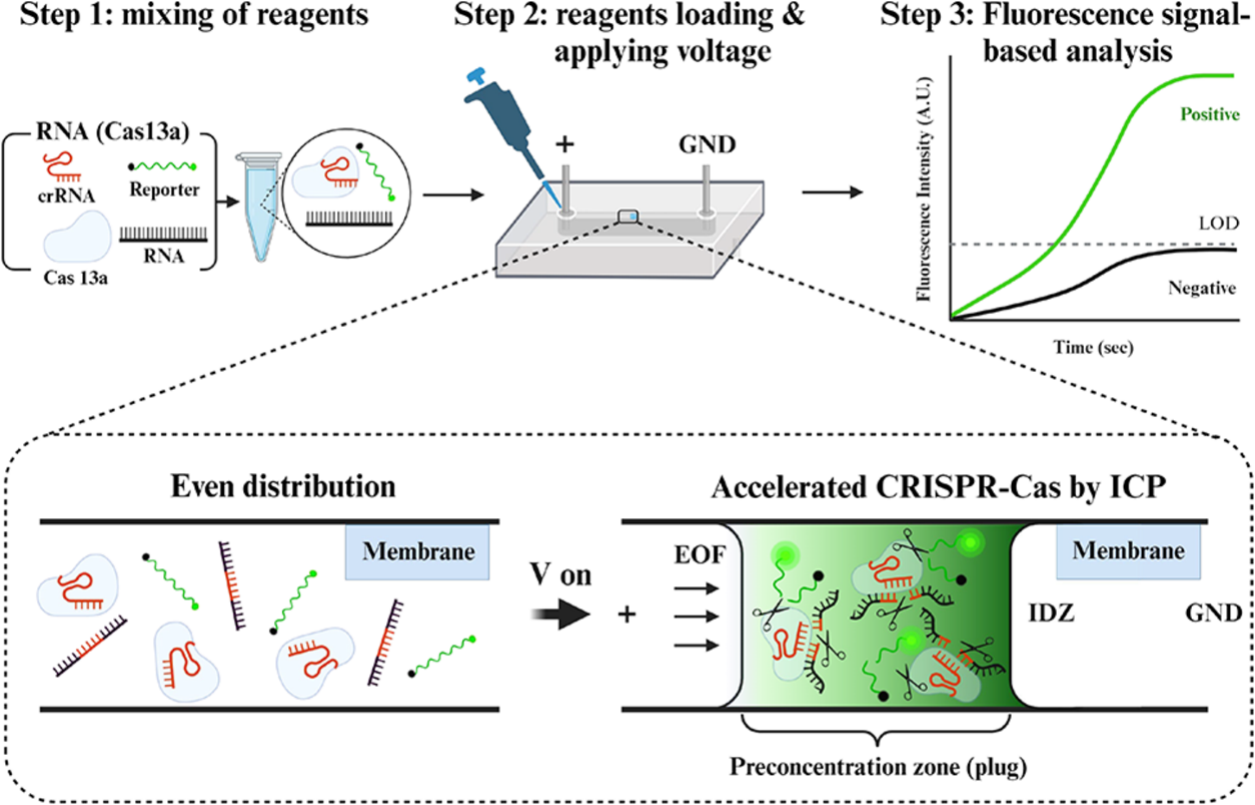
Detection of RNA using an ICP-based Microfluidic Accelerator Combined with CRISPR (IMACC). The IMACC workflow consists of three key steps: (1) Reagent Preparation: Cas13a, crRNA, reaction buffers, and target RNA are mixed. (2) Loading and ICP generation: the reagent mixture is loaded into the reservoir, and a voltage is applied across the microfluidic channel. This generates a preconcentration zone, visible as a focused plug near the IDZ, where CRISPR collateral cleavage is accelerated due to a shorter diffusion length, while cleaved fluorescence reporters accumulate inside the plug. (3) Detection and Analysis: the fluorescence signals of cleaved reporters are monitored and analyzed.

Nucleic acid detection scheme with IMACC involved three steps: (1) mixing and loading of CRISPR-based assay mixture including target RNA: miRNA-21 or SARS-CoV-2 RNA, a single type of Cas enzyme (i.e., Cas13a) with its pairing crRNA for each target, (2) applying voltage across the microfluidic channel to generate ICP and accelerate CRISPR-based collateral cleavage in the localized preconcentration zone of the single, straight microfluidic channel while simultaneously concentrating cleaved fluorescent reporters, and (3) detection and analysis of fluorescence signal generated, as shown in Figure 1.

When a voltage is applied across the microfluidic channel, cations preferentially pass through the cation-selective membrane, while anions are repelled, creating an ion imbalance that forms an ion depletion zone (IDZ) on the anodic side. Simultaneously, the Cas-crRNA complex (RNP), target RNA (miRNA-21 or SARS-CoV-2 RNA), and reporter molecules migrate toward the IDZ under the influence of the electroosmotic force (EOF). This movement leads to their accumulation in a localized preconcentration zone, forming a focused plug in front of the IDZ. Within this confined space, the diffusion length is reduced, while the concentration of cleaved reporter molecules increases, enhancing CRISPR-based nucleic acid detection.

To comprehensively understand ICP and RNA preconcentration, we conducted computational simulations using a well-tested house code^23^ by solving the Nernst–Planck, Poisson, Navier–Stokes, and RNA velocity equations. Simulations confirmed that ICP in the microchannel arises from electroosmotic flow (EOF_1_) and bulk flow, which drive ions and RNA toward the ion-selective membrane. The high membrane conductance creates an ion depletion zone (IDZ) with amplified electric fields, forming a vortex (EOF_2_) that prevents RNA leakage. These amplified fields trap RNA molecules, facilitating preconcentration in front of the IDZ with a maximum enrichment factor of 6.4 × 10^5^, as RNAs replace anions in the preconcentration region, as shown in Figures S2 and S3.

**Figure 2:**
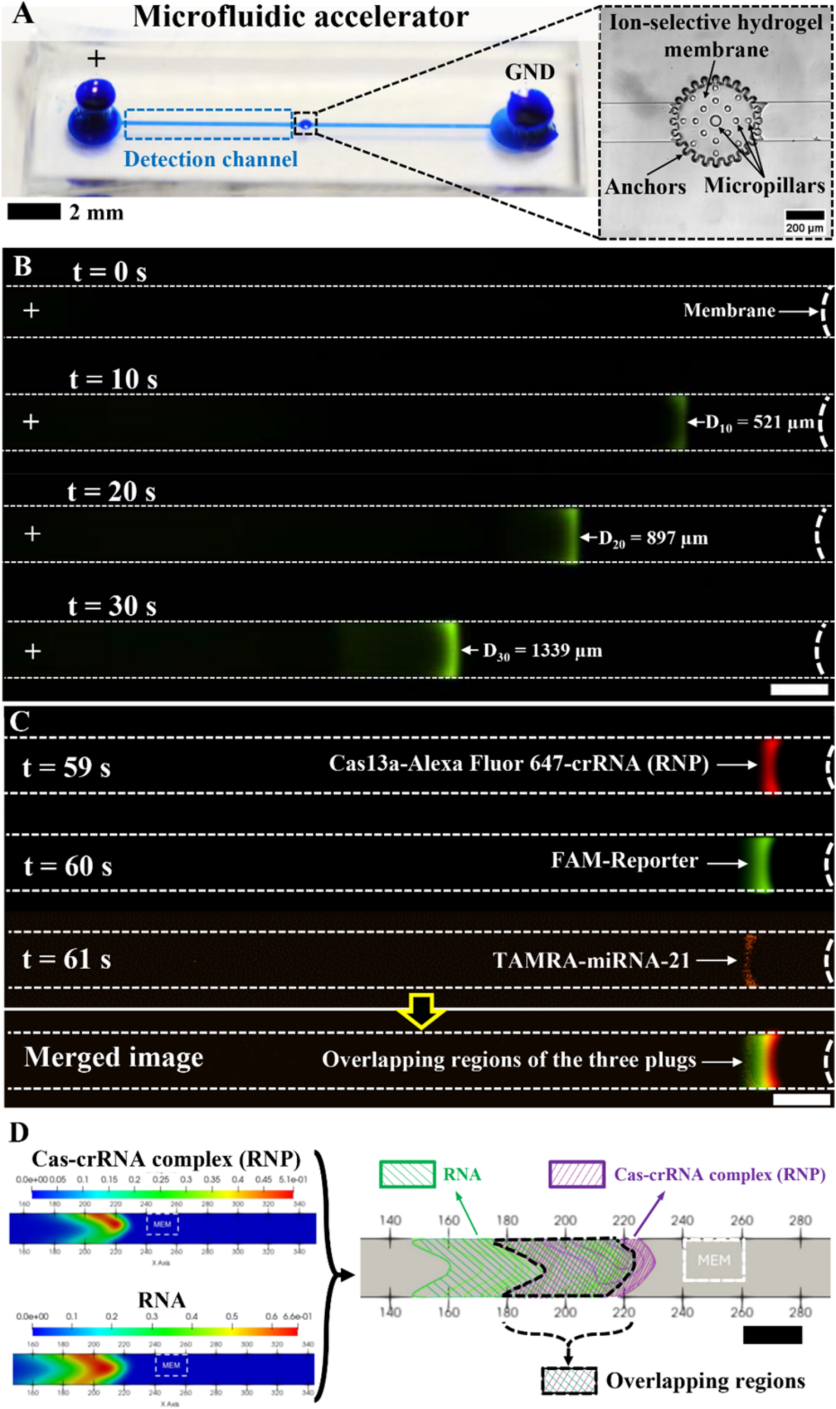
Microfluidic accelerator with PolyAMPS hydrogel-based ion-selective membrane and detection of RNA (e.g., miRNA-21) in IMACC. (A) A photograph showing a single and straight microfluidic channel of the microfluidic accelerator and an enlarged bright-field image showing a PolyAMPS hydrogel-based ion-selective membrane polymerized in the middle of the channel. (B) Fluorescence signal generated from FAM dye as a function of preconcentration time *t*, which was cleaved by Cas13a-crRNA complex (RNP) targeting miRNA-21 at *c* = 100 nM in IMACC at 60 V. At *t* = 10 s, the fluorescence signal became visible as a plug that slowly receded to the anodic reservoir at *t* = 20 and 30 s while its signal intensity continuously increased. The scale bar is 200 μm. (C) Validation of the overlapping location of each CRISPR reagent during detection of miRNA-21 via IMACC. The fluorescence image showed the location of each CRISPR reagent tagged with three fluorescence dyes: Ribonucleoproteins (RNP), which is a complex form of Cas13a-crRNA was observed as red color due to Alexa Fluor 647 tagged on crRNA, and the cleaved reporter was observed as green due to the FAM tag on the reporter, and the miRNA-21 was observed as orange due to the TAMRA tagged on miRNA-21. There was approximately a *t* = 1 s difference in the observation time for each CRISPR reagent due to the plug’s receding during the ICP and the time lag by the fluorescence filter change. The scale bar (white) is 200 μm. (D) A set of images shown on the left shows numerically studied preconcentration plugs of Cas-crRNA complex (RNP) and RNAs during ICP, and their merged 2D image with mesh, shown on the right, demonstrates overlapping of each plug between Cas-crRNA (RNP: green) and RNAs (purple), which is comparable with the result from experiments. MEM stands for a cation-selective membrane. Scale bar (black): 20 units of the *x*-axis equals 400 μm.

Figure 2A shows the IMACC microfluidic accelerator chip, featuring a single straight channel with an ion-selective hydrogel membrane at its center and two reservoirs serving as the anode (+) and cathode (GND). The loading volume is 10 μL, significantly smaller (5–10 times) than the 50–100 μL required for bulk CRISPR reactions. This minimized reagent and sample consumption is one of the key advantages of microfluidics over bulk assays. An enlarged bright-field image shows the structural features of the hydrogel membrane, which consists of a circular trench with multiple pillar anchors and cured PolyAMPS hydrogel completely filling the gaps. This design increases the surface interaction between the prepolymer solution and PDMS, enabling direct PolyAMPS deposition without chemical modification. The direct contact printing method ensures a reproducible, leakage-free hydrogel membrane, supporting stable ICP operation, as previously reported.^24^ During detection, fluorescence signals were monitored in the channel region adjacent to the left side of the membrane (blue dashed rectangle; Figure 2A).

Figure 2B shows time-lapse images of RNA detection (e.g., miRNA-21) via IMACC, with time *t* referring to preconcentration time by ICP. At *t* = 0 s, no fluorescence signal appeared in the anodic detection channel, as only diffusion-based CRISPR reactions occurred without ICP. Upon applying 60 V, a fluorescent plug became visible at *t* = 10 s, intensifying over time. Initially, the plug was narrow and expanded along the *x*-axis as the reaction time progressed. By estimating plug volume based on measured dimensions, the reaction volume started at ∼130 pL (area of the plug: *A* ≈ 6500 μm^2^, microchannel height: *H*_C_ = 20 μm) of the plug was generated at *t* = 10 s, increasing to ∼181 pL (*A* ≈ 9073 μm^2^) at *t* = 20 s and ∼224 pL (*A* ≈ 11,203 μm^2^) at *t* = 30 s. This suggests that IMACC physically confines reactions to picoliter volumes, effectively shortening the diffusion length and accelerating detection. Importantly, fluorescence intensity increased alongside plug expansion, indicating that collateral cleavage continuously produced cleaved reporters, accumulating within the confined space. As concentration time increased, the plug migrated toward the anodic reservoir, observed at *D*_10_ = 521 μm when *t* = 10 s (*D*_*n*_ = distance of the plug from the membrane at *t* = *n* seconds), then ∼1.7 times farther at *t* = 20 s, and ∼2.6 times farther at *t* = 30 s (white arrows), respectively. This time-dependent change of plug position was induced by expansion of IDZ during ICP^25^ and imbalance between electroosmotic flow and electrophoretic movements that lead to net velocity and direction of the plug toward the anode,^17^ necessitating its continuous tracking during imaging.

To validate the location of each CRISPR reagent during ICP in IMACC, we conducted a fluorescence-based observation to confirm the location of each CRISPR reagent. For this study, Alexa Fluor 647-crRNA, FAM-reporter, and TAMRA-miRNA-21 were used. As shown in Figure 2C, overlapping regions of the Cas13a-crRNA complex tagged with Alexa Fluor 647 (red), the cleaved reporter tagged with FAM (green), and miRNA-21 tagged with TAMRA (orange) were observed during the ICP, even though the time for the observation of each reagent was not identical for two reasons: continuous receding of the plug during the ICP and time lag due to change of the fluorescence filter. This result implied that our system allows effective shortening of the diffusion length of all CRPSR reagents in picoliter without significant dissociation.

To further investigate the location of each CRISPR reagent, numerical studies were conducted using the parameters of the Cas-crRNA complex (RNP)^20^ and RNAs.^26^Figure 2D exhibits numerically studied preconcentration plugs of Cas-crRNA complex (RNP) and RNAs formed by their accumulation in front of IDZ. As shown in the images on the left, both the Cas-crRNA complex (RNP) and RNAs gradually replaced all anions to neutralize with cations,^27^ eventually becoming plugs near the membrane by ICP. The merged 2D images with mesh mapping shown on the right demonstrated the overlapping of each plug between Cas-crRNA (RNP) and RNAs, which was comparable with the experimental result. The partial overlapping between the Cas-crRNA complex (RNP) and RNAs allowed shortening diffusion length between each reagent, which resulted in the acceleration of cleavage activity of CRISPR as well as preconcentration of the cleaved reporter during the ICP.

In short, we demonstrated the feasibility of RNA detection (e.g., miRNA-21) using IMACC without enzymatic amplification. The ICP-induced picoliter confinement effectively shortened the diffusion length, accelerating CRISPR cleavage activity while simultaneously accumulating cleaved fluorescent reporters. This synergy enabled rapid signal generation, allowing detection of the target RNA at high concentrations (*c* = 100 nM) within *t* = 30 s after ICP initiation.

### A Short-Length RNA: miRNA-21

To quantitatively assess IMACC’s detection capability, we tested miRNA-21, a known biomarker for early cancer and chronic disease diagnosis.^28^ After applying 60 V, all five synthetic samples at *c* = 10–10^5^ pM showed detectable fluorescence plugs within *t* = 30 s, whereas the negative control displayed only a weak signal (Figure S4A). Quantitatively analyzing the fluorescence signal over time confirmed that all synthetic samples from 10 to 10^5^ pM showed higher signal intensity than the negative control over time (Figure S4B). Detection of miRNA-21 down to 10 pM was achieved within 2 min (see Movie S1) due to a “compound” effect, which is likely based on the shortened diffusion length of each reagent accelerating CRISPR-Cas13 collateral cleavage while concentrating the cleavage reporter. The results demonstrated robust detection of miRNA-21 in IMACC without molecular amplification (e.g., PCR, RPA, LAMP). During the ICP, there might be a small change of 0.5 in pH, as previously reported.^24^ However, we believe that the activity of CRISPR remains stable since we observed stronger signal increases from all synthetic samples than from the negative control.

**Figure 3:**
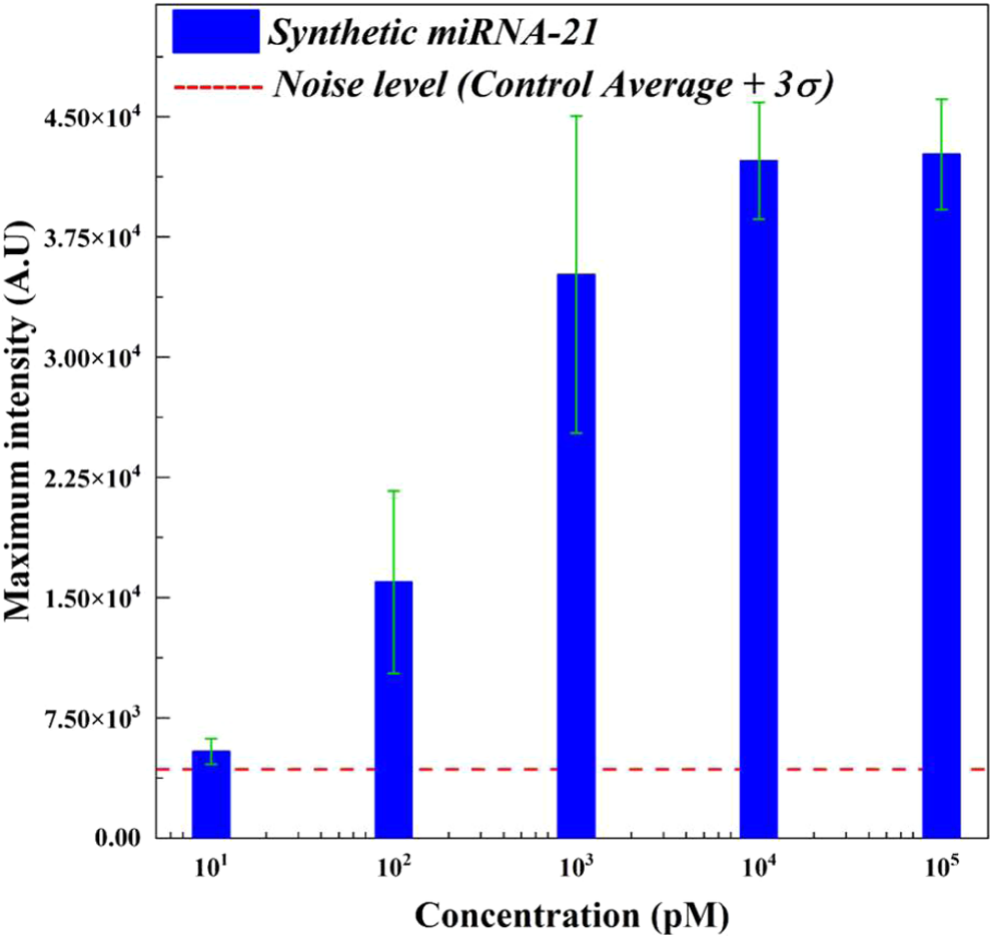
Quantitative analysis for detection of the synthetic miRNA- 21 using various concentrations from *c* = 10 to 10^5^ pM by IMACC at 60 V. The maximum intensity showed an increase as a function of the concentration of miRNA-21. All five concentrations showed maximum intensity values above the value of the noise level (control average +3σ: red dashed line).

To define the detection range, we further analyzed the maximum intensity and initial velocity *v*. The details of initial velocities *v* can be found in Figure S4C. Figure 3 shows that the maximum intensity at 10 pM exceeded the noise level (control average +3σ). These results indicated that there was the target recognition of Cas-crRNA (RNP) and collateral cleavage during ICP, which eventually allowed an increase in signal over the control. In other words, the 10 pM was detectable, even though the initial velocity *v* was not comparable to higher concentrations from *c* = 10^2^ to 10^5^. Based on this analysis, the current detection limit of IMACC for miRNA-21 is 10 pM, with a qualitative detection range of 10–10^5^ pM.

While some linearity was observed with increasing concentration, IMACC is currently a qualitative detection, given the aggregate aspects, such as noise superposition. The sensitivity of IMACC for miRNA-21 is lower than that of PCR, likely due to the small RNA size and its electrophoretic mobility, which limit ICP-enhanced detection. We believe IMACC’s sensitivity for short RNAs can be further enhanced by optimizing membrane conductivity through the addition of metallic ions or nanoparticles.^29^ Alternatively, dual-membrane configurations or electrically grounding the membrane could further improve performance, as predicted by computational simulations.^30^

For comparison, diffusion-based detection in a microchannel and 384-well plate was tested, as shown in Figures S5 and S6. Microchannel assays required *t* ≥ 500 s to detect signals at the highest concentrations (10^4^, 10^5^ pM), while lower concentrations remained undetectable, indicating lower sensitivity in the microchannel at the given observation time. In the microplate, all concentrations were eventually detected (*t* = 9000 s), but 10 pM required *t* ≥ 4000 s, confirming the slower detection speed of diffusion-based assays.

**Figure 4:**
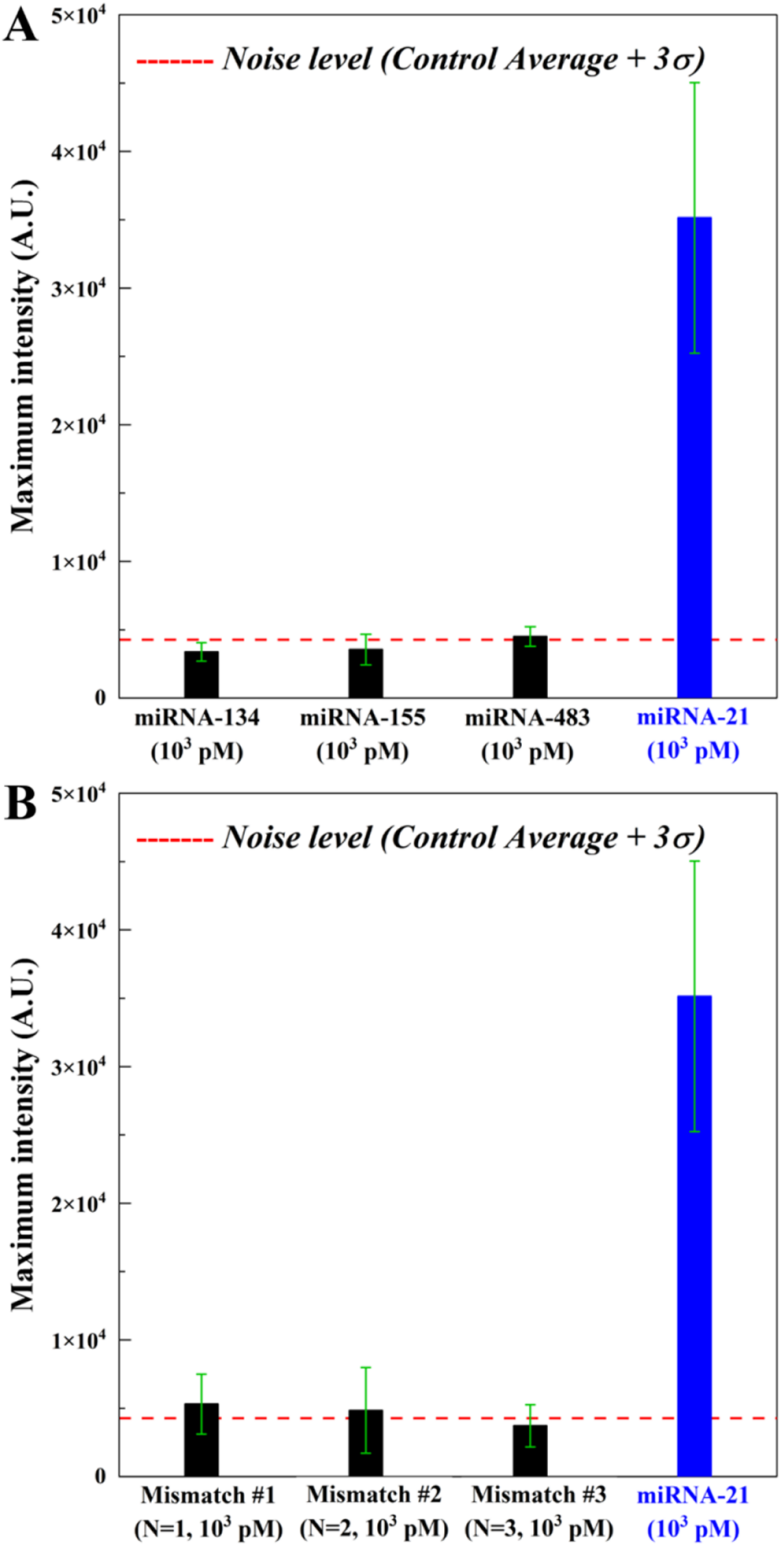
Investigation specificity of IMACC using off-target or mismatch sequences. (A) The maximum intensity obtained from all the off-targets (miRNA-134, 155, and 483: black bar), and from (B) different numbers of mismatches (*N* = 1, 2, and 3: black bar) were much less than that of the target (miRNA-21: blue bar), demonstrating specific detection of IMACC.

Next, the specificity of IMACC was tested using off-targets (miRNA-134, 155, 483) and mismatch sequences (N = 1, 2, 3) against the target, miRNA-21. The maximum intensity showed weaker signals for off-targets and mismatches, slightly above the noise level (control average +3σ) but far lower than the target, as shown in Figure 4. The initial velocity *v* from both off-targets and mismatch sequences also showed lower values than the target and similar values to the noise level (Figure S7). This confirms IMACC’s performance while maintaining CRISPR’s specificity to single mismatches (*N* = 1).^7^

In short, IMACC has enabled rapid and amplification-free detection of a short RNA, miRNA-21 within the detection range from *c* = 10 to 10^5^ pM in less than 2 min. IMACC could potentially be valuable for disease progression and prognosis, where specific concentration levels (up- or down-regulated) indicate different disease stages. Additionally, its simplified workflow and shortened detection time make it a promising tool for the rapid screening of diseases using miRNA-21 as a biomarker.

### A Long-Length RNA: SARS-CoV-2 RNA

We further validated IMACC for detecting long RNA (SARS-CoV-2 RNA) using serially diluted synthetic samples (*c* = 10^–1^ to 10^5^ copies/μL) without reverse transcription or amplification. As exemplified in Figure S8 and Movie S2, the signal increase from the synthetic samples (*c* = 10^0^ copy/μL) at the early stage was not significantly higher than the negative control, but after *t* = 120 s, a sharp increase in fluorescence was observed, while the negative control remained negligible. Figures S9 and S10 showed the signal generated from all synthetic samples at various concentrations *c* = 10^–1^–10^5^ copies/μL increased over time and became much brighter than the control after *t* = 70–120 s. To quantify IMACC’s performance in detecting SARS-CoV-2, initial velocities ν and maximum intensities of all synthetic samples and negative controls were analyzed (Figure S11). Unlike miRNA-21, the nonlinear trend was observed from both initial velocities and maximum intensities. As the copy number increased, the initial velocities and the maximum intensities kept decreasing and increasing back again.

To evaluate the applicability of IMACC to clinical samples, we tested 14 RT-qPCR-confirmed cases: 7 healthy and 7 SARS-CoV-2-infected patient samples (0.41–24,505.11 copies/μL). As shown in Figure S12, infected samples exhibited higher average intensities than healthy controls. However, similar to the synthetic samples, we observed the nonlinearity as a function of copy number and a large variation in both initial velocities (*ν*) and maximum intensities (Figure S13). Statistically, the maximum intensities analysis confirmed only a limited number of copy number cases (10^–1^ copy/μL of synthetic sample and 0.41, 0.80, 477.58, 11,597.14, and 24,505.11 copies/μL of infected patient samples) exceeded the noise level, while most lacked significance due to large variations in each value. Consequently, the IMACC system is currently limited to the detection of short RNAs such as microRNAs. Detecting long RNAs such as SARS-CoV-2 requires further process development in the future.

Compared to miRNA-21 results, these findings suggest a molecular size dependency in IMACC performance, potentially due to macromolecular crowding effects.^31^ Since Cas enzymes, allosteric enzymes are known to be sensitive to crowding,^32^ making this a plausible explanation. Particularly with large substrates like SARS-CoV-2 (∼30 kb, ∼9572 kDa), which could create a Crowded Milieu in the spatially confined ICP volume, leading to nonlinear correlations at high copy numbers. This thermodynamic nonideality seems similar to the study showing decreased activity at high concentrations of substrates, explained by expansion and an increase in asymmetry of the ES complex during catalysis.^33^ In addition, the target site in the N gene (20 bp) constitutes only 0.067% of the total length of SARS-CoV-2 RNA, which may hinder recognition of the target site, further slowing reaction rates in the preconcentration zone due to crowded conditions. In contrast, miRNA-21, being much shorter, has a smaller radius of gyration, making the crowding effect less pronounced.^34^ Additionally, ICP is a complex process influenced by concentration gradients and electro-convection, beyond simple diffusion,^17^ suggesting that both molecular crowding and ICP’s nonlinear kinetics contribute to the lack of direct correlation between copy number and fluorescence intensity. Another possible explanation is electric field-mediated dissociation between Cas and RNA.^35^ All of these hypotheses, however, require a further in-depth investigation for validation.

IMACC shows strong potential as a rapid screening tool for RNA detection, even though it is currently quantitative. From the point of view of analysis, a single criterion, the maximum intensity, is sufficient for qualitative detection. Potentially, there is room for improvement by changing the composition and reaction ratio between the Cas13a enzyme and crRNA for improving performance. Controlling this reaction parameter could enable a quantitative detection on IMACC. In addition, the minimization of receding of the plug by increasing the viscosity of the solution can increase the residence time of the reaction, potentially enabling quantitative detection with reliability. Further future improvement could include consolidating the IMACC assay onto a single microfluidic device along with sample preparation steps as well as an automatic detection system. Such an integrated automatic sample-to-readout nucleic acid detection platform based on ICP and CRSIPR can be a powerful tool for diagnostics.

## Conclusions

In conclusion, IMACC enables the rapid detection of RNA (miRNA-21) by integrating CRISPR-Cas13 with ICP in a microfluidic accelerator device. Designed as a single-channel system with a directly deposited membrane in PDMS, IMACC is easy to fabricate and operate. A preconcentration zone formed by ICP, allowing overlapping between target RNA and RNP, resulting in shortened diffusion length, thereby accelerating CRISPR-Cas13 collateral cleavage while concentrating cleaved reporters within a physically confined space. IMACC enables the rapid detection of miRNA-21 at concentrations as low as 10 pM within 2 min while ensuring CRISPR specificity during ICP, effectively minimizing false signals from off-target and single-base mismatches (*N* = 1) sequences. Currently, IMACC is a qualitative detection and is limited to the detection of short RNAs such as microRNAs. Detecting long RNAs such as SARS-CoV-2 requires further process development in the future. However, its simplified detection process, high sensitivity, and fast turnaround time hold promise for taking CRISPR-based diagnostics to the next level. Furthermore, IMACC holds potential flexibility for the detection of various diseases by simply reprogramming crRNA for desired targets without altering the device setup. This makes it a promising, versatile platform for detecting infectious diseases as well as liquid biopsy-based cancer detection, cardiovascular diseases, and pathogen identification targeting DNA, RNA, or rRNA biomarkers.

## Supporting Information Available

Additional experimental details, materials, and methods, including sequences, composition and volume of each reagent used for IMACC, a schematic of a microfluidic accelerator, additional data from detection of RNA targets from both synthetic and clinical samples using IMACC, boundary conditions for numerical methods (PDF)
Movie S1: the detection of miRNA-21 at *c* = 10 pM in IMACC (MP4)
Movie S2: the detection of SARS-CoV-2 at *c* = 10^0^ copy/μL in IMACC (MP4)
Movie S3: the detection of SARS-CoV-2 at *c* = 10^–1^ copy/μL in IMACC (MP4)
