# Supplementary material for: Accelerating Cleavage Activity of CRISPR-Cas13 System on a Microfluidic Chip for Rapid Detection of RNA
Source: Anal Chem. 2025 Apr 30;97(18):9858–65. doi: 10.1021/acs.analchem.5c00256 (PMC12079638; doi:10.1021/acs.analchem.5c00256)
Supplement: Supplementary file 1 — ac5c00256_si_001.pdf [file ac5c00256_si_001.pdf]

Supporting Information

Accelerating Cleavage Activity of CRISPR-Cas13 System on a Microfluidic Chip for Rapid Detection of RNA

Jongmin Kim[a], Ajymurat Orozaliev[a], Sarah Sahloul[a], Anh-Duc Van[a, b], Van-Truong Dang[c], Van-Sang Pham[c], Yujeong Oh[d], Ibrahim Chehade[d], Mohamed Al-Sayegh[d] and Yong-Ak Song[a, e, f, *]

[a]Division of Engineering, New York University Abu Dhabi, PO Box 129188, Abu Dhabi, UAE

[b]Department of Mechanical and Aerospace Engineering, Tandon School of Engineering, New York University, New York 11201, USA

[c]School of Mechanical Engineering, Hanoi University of Science and Technology, No. 1 Daicoviet Road, Hanoi, Vietnam

[d]Division of Science, New York University Abu Dhabi, PO Box 129188, Abu Dhabi, UAE

[e]Department of Chemical and Biomolecular Engineering, New York University Tandon School of Engineering, Brooklyn, NY 11201, USA

[f]Department of Biomedical Engineering, New York University Tandon School of Engineering, Brooklyn, NY 11201, USA

[*]Correspondence: rafael.song@nyu.edu; Tel.: +971-2-628-4781

Table of Contents

1. **Materials**
2. **Experimental conditions**
3. **Supporting Tables**

   Table S1 All sequences used for IMACC, including crRNAs, reporters, targets, off-targets, and mismatches.

   Table S2 The composition and volume of each reagent used for IMACC.
4. **Supporting Figures**

   Figure S1. A schematic showing detailed dimensions of a microfluidic accelerator.

   Figure S2. Simulation results of ICP at c = 1 mM of ion concentrations solution.

   Figure S3. Numerically studied preconcentration of RNA.

   Figure S4. Characterization and quantitative analysis for detection of the synthetic miRNA-21 using various concentrations from c = 10 pM to 10^5 pM by IMACC at 60 V.

   Figure S5. Detection of miRNA-21 with varied concentration c = 10 pM to 10^5 pM in the microfluidic channel based on diffusion only without ICP as a control experiment.

   Figure S6. Detection of miRNA-21 with varied concentration c = 10 pM to 10^5 pM in a 384-microwell by using a microplate reader as a conventional approach.

   Figure S7. Investigation specificity of IMACC using off-targets or mismatch sequences.

   Figure S8. Time-lapse image showing fluorescence signal change of plugs generated from negative control and SARS-CoV-2 at c = 10^0 copy/µL in IMACC.

   Figure S9. Observation of signal and quantitative analysis for detection of SARS-CoV-2 RNA by IMACC at the four representative copy numbers starting from c = 10^{-1} to 10^2 copies/µL.

   Figure S10. Detection of synthetic SARS-CoV-2 RNA at various concentrations c = 10^{-1} to 10^5 copies/µL using IMACC.

   Figure S11. Quantitatively analyzed initial velocity v and maximum intensity computed from intensities of synthetic SARS-CoV-2.

   Figure S12. Detection of SARS-CoV-2 using IMACC from clinical samples.

   Figure S13. Quantitatively analyzed initial velocity v and maximum intensity computed from intensities of SARS-CoV-2 in clinical samples.
5. **Supporting Movies**

   Movie S1.

   Movie S2.

   Movie S3.
6. **References**

1. Materials

Chemicals for microfabrication and hydrogel. SU-8 3025 (photoresist) and SU-8 developer were purchased from MicroChem (Newton, MA, USA). Poly(dimethylsiloxane) (PDMS) oligomer and curing agent was purchased from Dow Corning (Midland, MI, USA). 2-Acrylamido-2-methylpropane sulfonic acid (AMPS), N,N'-Methylenebisacrylamide (MBAm), (2-Hydroxy-1-(4-(2-hydroxyethoxy)phenyl)-2-methylpropan-1-one, also known as Irgacure 2959), DMSO (Dimethyl Sulfoxide) and Isopropyl alcohol (IPA) were purchased from Sigma-Aldrich (MO, USA). All chemicals were used as purchased without further purification. Deionized water (>18 MΩ·cm) was obtained from a Milli-Q water system (Milford, MA, USA).

Target nucleic acids and CRISPR reagents. A solution (100 μL, $c = 10^6$ copies/μL) of synthetic SARS-CoV-2 RNA control (29,865 bp) covering 99% of whole genomes of SARS-CoV-2 (102019 (MT007544.1)) was purchased and used as received from Twist Bioscience. Lyophilized synthetic miRNA-21 was purchased from Integrated DNA Technologies (IDT). Lyophilized synthetic miRNA-134, 155 and 483, and mismatch sequences (N = 1, 2, and 3) were purchased from Synbio Technologies. LbuCas13a (Cat. No. CS13A-E322U), 1X Cas13 storage buffer (Cat. No. CS13-19), 10X Cas13 reaction buffer (Cat. No. CS13-09) were purchased from SignalChem. A solution of RNase Inhibitor, Murine (M0314) was purchased from New England Biolabs (NEB). Custom-designed crRNA for miRNA-21, crRNA for N gene targeting in SARS-CoV-2 RNA, and Poly U reporter (single strand RNA) were purchased from Synbio Technologies.

2. Experimental conditions

Imaging and analysis. Fluorescence signals were captured using a Nikon Ti-Eclipse inverted epi-fluorescence microscope (Nikon, Tokyo, Japan) with an X-Cite XYLIS broad-spectrum LED illumination system (Excelitas, Waltham, MA, USA) and an Andor iXon Ultra DU-897U-CS0 EMCCD digital camera (Andor Technology, Belfast, Northern Ireland). Fluorescence images and videos were acquired under a FITC filter (λ_{em} = 520 nm) with a 300 ms exposure time. Image acquisition and intensity measurements were performed using NIS-Elements AR software and NIH ImageJ.

3. Supporting Tables

Table S1 All sequences used for IMACC, including crRNAs, reporters, targets, off-targets, and mismatches

Entry	Sequences (5'-3')
crRNA for miRNA-21[1]	GACCACCCCAAAAAUGAAGGGGACUAAAACAUCAACAUCAGUCUGAUAA<br>GCUA
crRNA for N gene of SARS-CoV-2 RNA[1]	GACCACCCCAAAAAUGAAGGGGACUAAAACAGGUCCACCAAACGUAAUG<br>CG
Poly U reporter	FAM-UUUUU-BHQ1
miRNA-21 (5p)	UAGCUUAUCAGACUGAUGUUGA
miRNA-134 (5p)[2]	UGUGACUGGUUGACCAGAGGGG
miRNA-155 (5p)[2]	UUAAUGCUAAUCGUGAUAGGGGUU
miRNA-483 (5p)[2]	AAGACGGGAGGAAAGAAGGGAG
Mismatch #1 (N = 1)[3]	UAG**G**UUAUCAGACUGAUGUUGA
Mismatch #2 (N = 2)[3]	UAGC**A**UAUCA**C**ACUGAUGUUGA
Mismatch #3 (N = 3)[3]	UAGCUUA**A**CA**C**ACUG**U**UGUUGA
N gene in SARS-CoV-2 RNA[4]	AUGUCUGAUAAUGGACCCCA AAAUCAGCGAAAUGCACCC**CGCAUUACG**<br>**UUUGGUGGACC**CUCAGAUUCAACUGGCAGUAACCAGAAUGGAGAACGC<br>AGUGGGGCGCGAUCAAAACAACGUCGGCCCCAAGGUUUACCCAAUAAUA<br>CUGCGUCUUGGUUCACCGCUCUCACU CAACAUGGCAAGGAAGACCUUA<br>AAU UCCCUCGAGGACAAGGCGUUCCAAUUAACACCAAUAGCAGUCCAG<br>AUGACCAAAUUGGCUACUACCGAAGAGCUACCAGACGAAUUCGUGGUG<br>GUGACGGUAAAAUGAAAGAUCUCAGUCCAAGAUGGUAUUUCUACUACC<br>UAGGAACUGGGCCAGAAGCUGGACUUCCCUAUGGUGCUAACAAAGACG<br>GCAUCAUAUGGGUUGCAACUGAGGGAGCCUUGAAUACACCAAAAGAUC<br>ACAUUGGCACCCGCAAUCCUGCUAACAAUGCUGCAAUCGUGCUACAACU<br>UCCUCAAGGAACAACAUUGCCAAAAGGCUUCUACGCAGAAGGGAGCAGA<br>GGCGGCAGUCAAGCCUCUUCUCGUUCCUCAUCACGUAGUCGCAACAGUU<br>CAAGAAAUUCAACUCCAGGCAGCAGUAGGGGAACUUCUCCUGCUAGAAU<br>GGCUGGCAAUGGCGGUGAUGCUGCUCUUGCUUUGCUGCUGCUUGACAG<br>AUUGAACCAGCUUGAGAGCAAAAUGUCUGGUAAAGGCCAACAACAACA<br>AGGCCAAACUGUCACUAAGAAAUCUGCUGCUGAGGCUUCUAAGAAGCCU<br>CGGCAAAAACGUACUGCC AC UAAAGCAUACAAUGUAACACAAGCUUUC<br>GGCAGACGUGGUCCAGAACAAACCCAAGGAAAUUUUGGGGACCAGGAA<br>CUAAUCAGACAAGGAACUGAUUACAAACAUUGGCCGCAAAUUGCACAA<br>UUUGCCCCCAGCGCUUCAGCGUUCUUCGGAAUGUCGCGCAUUGGCAUGG<br>AAGUCACACCUUCGGGAACGUGGUUGACCUACACAGGUGCCAUCAAAUU<br>GGAUGACAAAGAUCCAAAUUUCAAAGAUCAAGUCAUUUUGCUGAAUAA<br>GCAUAUUGACGCAUACAAAACAUUCCCACCAACAGAGCCUAAAAAGGAC<br>AAAAAGAAGAAGGCUGAUGAAACUCAAGCCUUACCGCAGAGACAGAAG<br>AAACAGCAAACUGUGACUCUUCUUCCUGCUGCAGAUUUGGAUGAUUUC<br>UCCAAACAAUUGCAACAAUCCAUGAGCAGUGCUGACUCAACUCAGGCCU<br>AA

[1]All sequences are composed of scaffold(constant) region and target region
[2]Used as off-targets
[3]Bold with underline indicates the location and sequence of mismatch as compared to the target, miRNA-21
[4] Bold with an underlined sequence is the target region by crRNA for N gene of SARS-CoV-2 control covering 99% of whole genomes of SARS-CoV-2 (102019 (MT007544.1))

Table S2 The composition and volume of each reagent used for IMACC

Entry	Reagents	Stock concentration	Volume/ 1 reaction (μL)	Final concentration[a]	Volume for N reactions (μL)
CRISPR master mix	Nuclease free water	-	3.75	-	3.75 × (N+1)[b]
	Cas13a reaction buffer	10X	1	1X	1 × (N+1)
	LbuCas13a	500 nM	1	50 nM	1 × (N+1)
	crRNA[c]	500 nM	1	50 nM	1 × (N+1)
	Poly U reporter	4 μM	1	400 nM	1 × (N+1)
	RNase inhibitor	40 U/μL	0.25	1 U/μL	0.25 × (N+1)
Nucleic Acids (NA) Targets	miRNA-21	10 pM-10^{5} pM	2	-	-
	SARS-CoV-2 RNA	10^{-1}-10^{5} copies/μL	2	-	-
Test sample	CRISPR master mix + a target	-	10	-	-

[a]The final concentration in 10 μL of the test sample, prepared by mixing 8 μL of CRISPR master mix and 2 μL of a target.
[b]N is a number of experiments, including all numbers of positive and negative samples and replicates. A master mix of volume N+1 was prepared to account for unexpected wastage that may occur during preparation and use.
[c]It is used either crRNA for miRNA or crRNA for N gene of SARS-CoV-2, respectively.

4. Supporting Figures

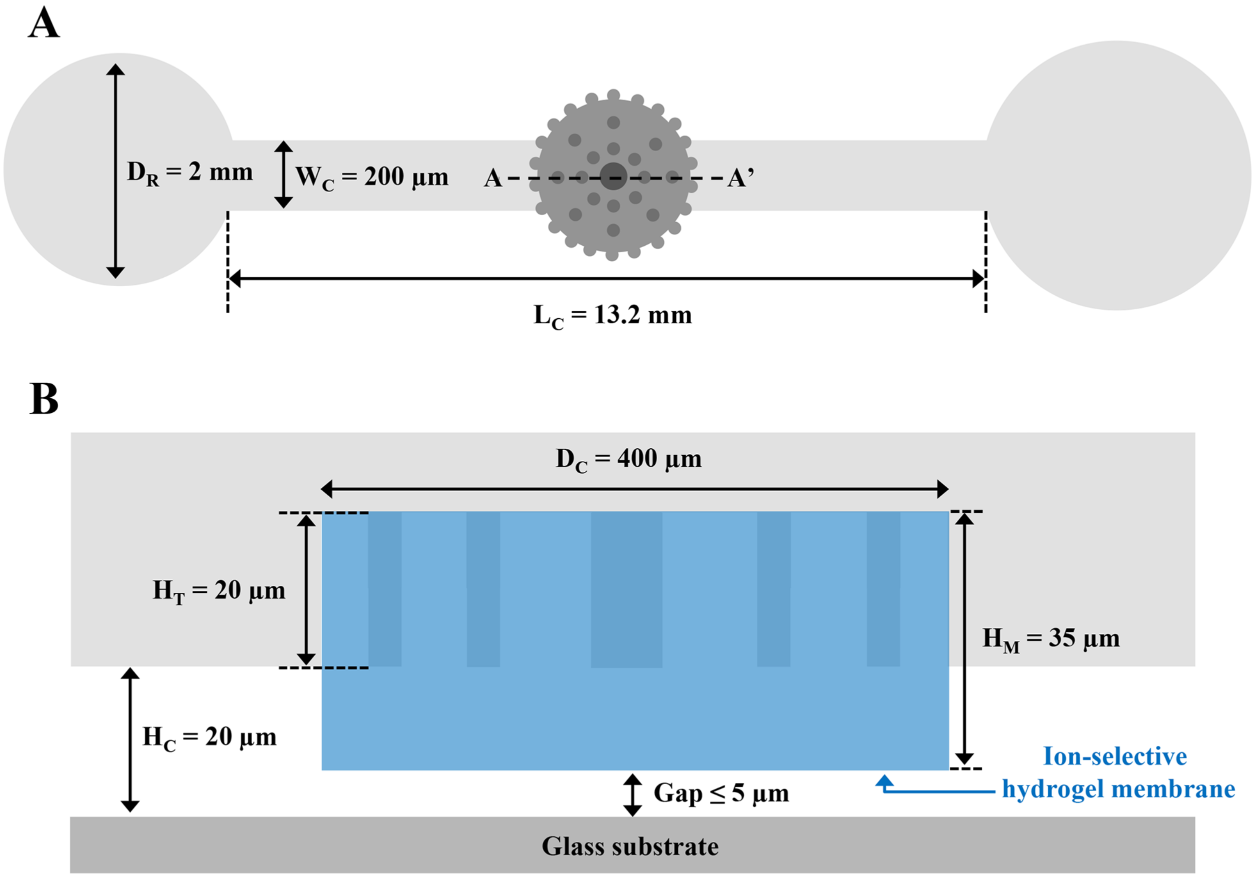

Figure S1. A schematic showing detailed dimensions of a microfluidic accelerator. (A) Top view of a microfluidic accelerator showing overview of structure and dimensions of length and width. (B) Cross-sectional view at the local area of A-A’ showing detailed structures and dimensions of circular pattern.

Device Fabrication

A microfluidic accelerator consisting of a single straight microfluidic channel with an integrated cation-selective hydrogel membrane was built via conventional soft lithography and UV-induced photopolymerization. Briefly, the PDMS microchannel ($H \times W \times L = 20\ \mu m \times 200\ \mu m \times 13.2$ mm) enclosed a circular trench in the middle containing micropillars placed inside as well as with multiple anchors on the circumference to enhance the attachment of the hydrogel membrane to the trench. Particularly, the circular trench where the hydrogel is intended to be formed was designed

to have a higher height (H_T = 40 μm) than the channel (H_C = 20 μm), as shown in **Figure S1**. After punching both the inlet and outlet holes (circular holes of 2 mm in diameter on each side), the PDMS device was placed on a blank glass substrate with the channel facing down, and plasma treated at 800 mTorr for 1 minute to surface activate the inner microchannel.

Next, the prepolymer solution composed of 1.5 M of AMPS as a monomer, 0.86 M of MBAm as a crosslinker (the molar ratio between AMPS : MBAm = 1.75 : 1), 1 wt% (to the mass of AMPS) of (2-Hydroxy-1-(4-(2-hydroxyethoxy) phenyl)-2-methylpropan-1-one) as a photoinitiator in DMSO was prepared. Then, the prepolymer solution was purged under N_2 gas atmosphere for 5 minutes, followed by mixing with IPA at a volumetric ratio of 8:2. This purged and diluted prepolymer solution was contact printed on the circular trench at the center of the PDMS microchannel using a fluidic dispensing system (microplotter II, Sonoplot Inc., Middleton, Wisconsin, USA).

The dispensing tip with an opening diameter of approximately 400 μm moved around the circular trench to fill it completely. The loaded prepolymer solution on the PDMS chip was cured by using a UV curing machine (OmniCure AC475-365 nm, Jenton International, Whitchurch, Hampshire, UK) at 365 nm for 6 minutes under N_2 gas atmosphere, forming a PolyAMPS-based cation-selective hydrogel membrane in the PDMS chip.

Finally, the accelerator was cleaned by repeating the washing process twice, which consisted of gentle drop-casting of deionized water onto the hydrogel, incubating for 4 minutes, followed by removing the water, then stored at room temperature before use. The final thickness of the cured hydrogel was approximately 35 μm protruding ~15 μm from the PDMS trench. We prepared the hydrogel membrane one day in advance and ran triplicate experiments for each

concentration/copy number. We used one device for one single experiment. Therefore, all the data contained batch-to-batch variation.

Note that the pre-prepared device was treated with plasma at 800 mTorr for 1 minute before use every time, which introduced hydrophilic moieties and negative charges that prevent significant nonspecific binding of targets or CRISPR-related molecules. As nucleic acid targets and Cas enzyme-crRNA complexes are negatively charged in the reaction buffer, nonspecific interactions are unlikely. Potentially, two additional approaches can be considered, such as coating the channel walls with SDS surfactant[1] or incorporating F127 (PEO-PPG-PEO block copolymer) during PDMS preparation.[2]

Numerical simulation

The ICP phenomena and the mechanism of preconcentration of RNAs were elucidated using the computational approach based on well-tested house code[3] by solving Nernst Planck equations (1), (2), Poisson equations (3), Navier-Stokes (4), (5), and the velocity equation of RNA (6), (7), (8), as follows:

$$\frac{\partial C_i}{\partial t} = -\nabla . J_i \quad (1)$$

$$J_i = -(D_i \nabla C_i + \mu_i Z_i C_i \nabla \phi) + UC_i \quad (2)$$

$$\rho_e = F \sum Z_i C_i = -\varepsilon_o \varepsilon_r \nabla^2 \phi \quad (3)$$

$$\nabla . U = 0 \quad (4)$$

$$\rho \frac{\partial U}{\partial t} = -\nabla P + \eta \nabla^2 \mathrm{U} - \rho(\mathrm{U}.\nabla)\mathrm{U} - \rho \nabla \phi \quad (5)$$

$$U_{RNA} = U + U_{EP} \quad (6)$$

$$U_{EP} = \mu_{RNA} E \tag{7}$$

$$E = -\nabla \phi \tag{8}$$

Where C_i, J_i, D_i, μ_i, Z_i are concentration, ionic flux density, diffusion coefficient, the electrophoretic mobility, and ion valence of an ion species i-th. U, P, ρ, η denote the velocity, pressure, density, and dynamic viscosity of the solution. t is time, Φ is the electrical potential, E is electrical field. Other parameters related to RNA include electrophoretic mobility μ_{RNA}, RNA's velocity U_{RNA}, electrophoretic velocity U_{EP}. The buffer solution is 1 mM potassium chloride (KCl) with a diffusion coefficient of K+ and Cl- are $D_1 = 1.957\times10^{-9}$ m^2s^{-1} and $D_1 = 2.032\times10^{-9}$ m^2s^{-1} respectively. RNAs were considered as negatively charged molecules with electrophoretic mobility $\mu_{RNA} = 2.91\times10^{-8}$ m^2s^{-1}.[4]

In the simulation model, we scaled down the microchannel and membrane in the x-direction to lengths of 500 µm and 20 µm, respectively, and in the y-direction to heights of 20 µm for the channel and 15 µm for the membrane. A hexahedral mesh with refining regions near the membrane and microchannel walls was used. This configuration allows us to reduce computational costs while still accurately capturing the preconcentration of RNA as the concentration profile across the channel. In the vicinity of the cation-selective membrane, the RNA concentration showed a peak. The boundary conditions include:

1) At the inlet and outlet, a pressure difference of 0.94 Pa between the inlet and outlet represented the height difference of 96 µm between the two reservoirs. A voltage of 2.26 V across the boundaries maintained the electric field of 4.5 V/mm, which was used in our experiments.
2) A zeta potential of −20 mV was applied at the microchannel walls, with a no-slip condition for velocity and no flux for ions and RNA.

3) In the cation-selective membrane domain, a fixed negative charge was assumed to be distributed uniformly throughout the membrane area.

Figure S2 shows the simulation results of ICP phenomena within the microchannel. Under the influence of the electric field on the electric double layer near the channel wall, a fluid flow is generated, moving from the anode side to the cathode side. This flow is called electroosmotic convection, or the first kind of electroosmotic flow (EOF_1). The combination of EOF_1 and the pressure-driven flow called the bulk flow, drags cations, anions, and RNAs toward the ion-selective membrane. Due to the membrane conductance (J_σ) being significantly greater than the bulk conductance (J_{bulk}), the Dukhin number ($Du = J_\sigma/J_{bulk}$) is extremely high, causing the preference of cations to go through the membrane rather than the bulk solution.[5] In the meantime, the anions and RNAs are repelled from the ion-selective membrane by the electrostatic repulsion of the membrane. Therefore, both cations and anions are significantly reduced at the front of the ion-selective membrane, generating the ICP phenomenon in the single channel, as shown in **Figure S2A**. The concentration of cations and anions is dropped from 1 mM to 0.06 mM at a steady state in the region near the membrane on the left side, forming an ion-depletion zone (IDZ). In the meantime, the lack of ions (the reduction of the electric conduction) in IDZ magnifies the electric field significantly up to 2E0 which is nearly 33 times higher than the average electric field in the bulk region (0.06E0), as shown in **Figure S2B**.

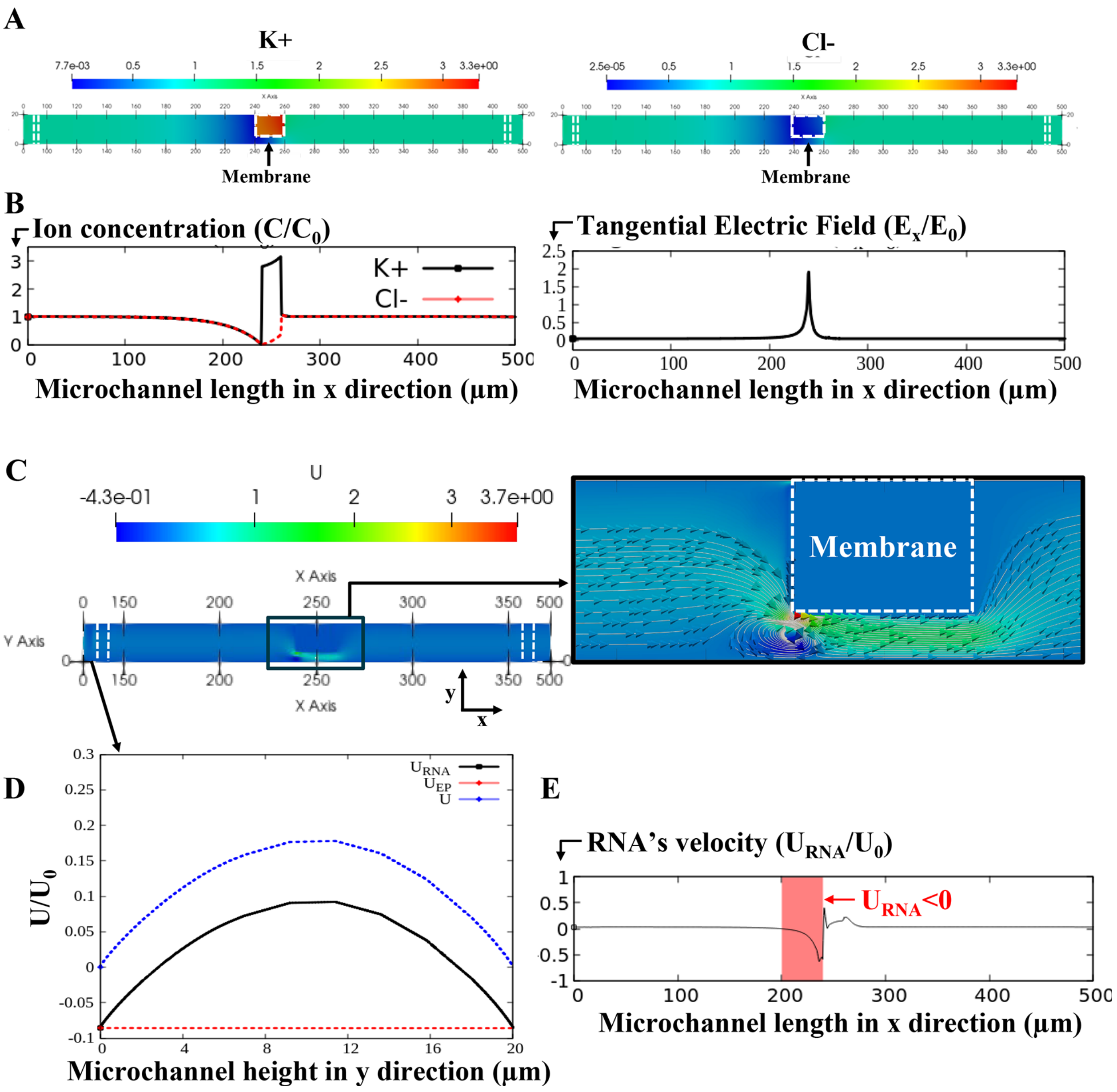

Figure S2. Simulation results of ICP at c = 1 mM of ion concentrations solution. (A) The formation of the ion-depletion region on the left side (Anode side) and the ion-enrichment region on the right side (Cathode side) of the membrane, as well as the concentration distribution of ions in microchannel. The vertical white dash lines indicate the omission of regions that exhibit uniform distribution. (B) The ion concentrations profile and electric field along the channel (y = 4.5 μm). (C) The simulation result of the flow field in the channel and enlarged image show the formation of a vortex called the second kind of electroosmotic flow (EOF_2) near the membrane. (D) Velocity profiles of flow (U), electroosmotic (U_{EP}), and RNA (U_{RNA}) along y direction near the inlet of the microchannel (x = 5 μm). (E) The profile of RNA's velocity along the x-direction (y = 4.5 μm), with the red region indicating where the velocity is less than 0.

These amplified electric fields in IDZ lead to electrodynamic instability of the fluid, resulting in the formation of a vortex called the second kind of electroosmotic flow (EOF_2) near the membrane, as shown in **Figure S2C**. The strength of the EOF_2 is proportional to the square of the electric field strength.[6] The fluid dynamics and the electric field in IDZ are the two crucial factors in generating the preconcentration of RNAs in the microchannel. The magnified electric field in IDZ reacts as a barrier to prevent RNAs from leaking toward the anode side of the channel while RNAs move from the channel inlet to the ion-selective membrane by EOF_1, as shown in **Figure S2D**. As a result, the net velocity of RNAs becomes negative and the RNAs get accumulated in red region, as shown in **Figure S2E**.

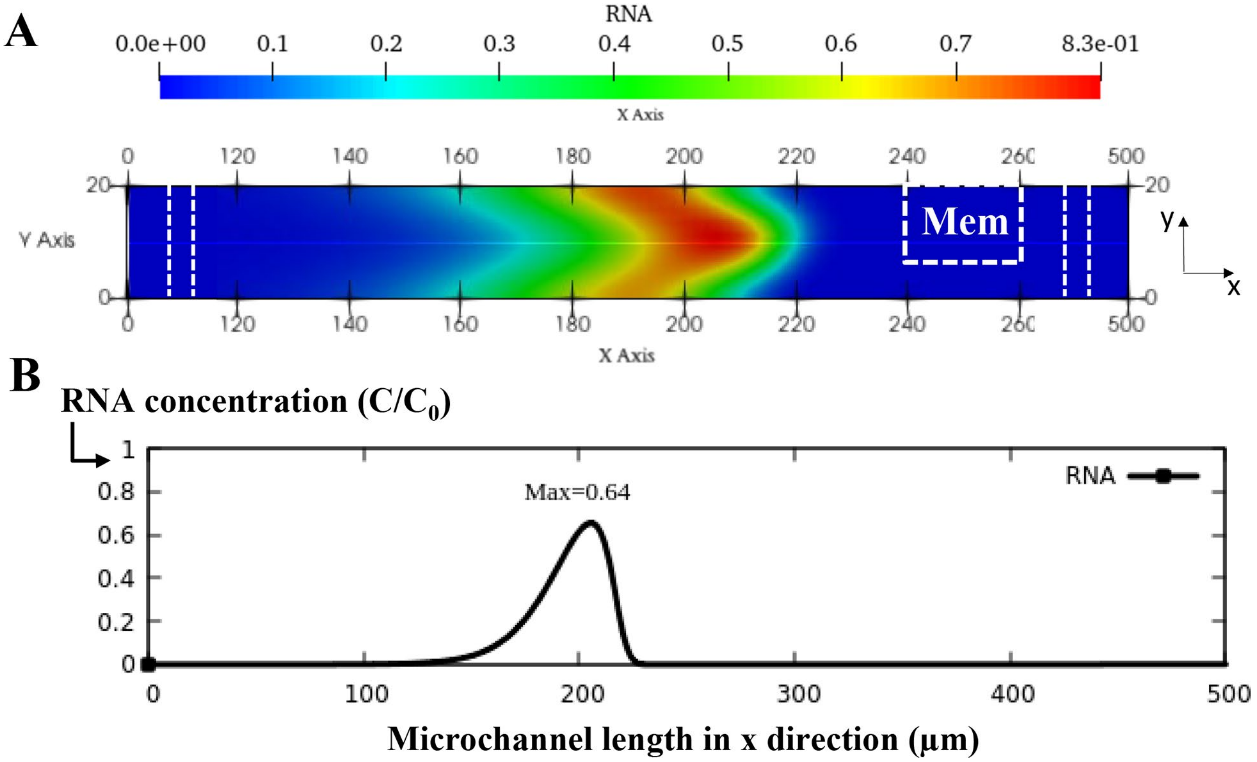

Figure S3. Numerically studied preconcentration of RNA. (A) Numerically studied the preconcentration plug of RNAs at c = 1 mM of ion concentration in IMACC (Mem = membrane, the vertical white dash lines indicate omission of regions exhibiting uniform distribution (blue)). (B) Numerically studied RNA concentration distribution along x direction (y = 10 µm).

Figure S3A exhibits numerically studied preconcentration plugs of RNAs formed by their accumulation in front of IDZ, where the maximum enrichment factor (EF) is located at x = 200 µm. In the preconcentration region, RNAs gradually replace all anions to neutralize with cations, so this maximum EF value is calculated by the ratio of the maximum RNA concentration in the channel (0.64 C0) to the initial RNA concentration value (1e-6 C0), resulting in 6.4e5, shown in **Figure S3B**.

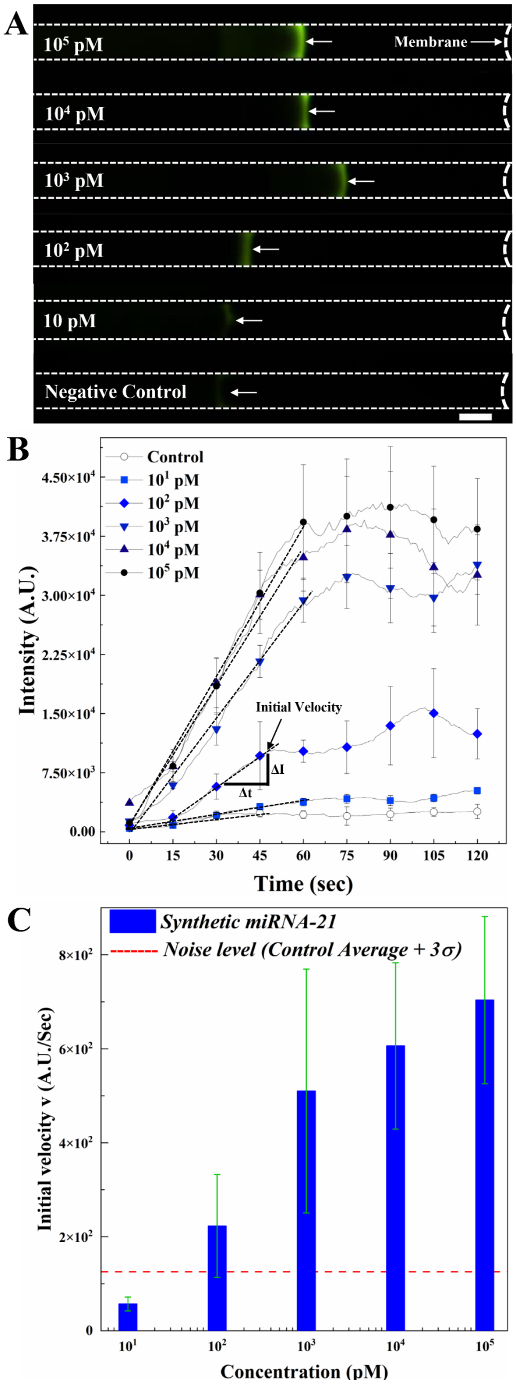

Figure S4. Characterization and quantitative analysis for detection of the synthetic miRNA-21 using various concentrations from c = 10 pM to 10^5 pM by IMACC at 60 V. (A) Fluorescence images showing generated signal at t = 30 s by detection of miRNA-21 using various concentrations starting from c = 10 pM to 10^5 pM. Scale bar = 200 μm (B) A graph showing quantitative analysis of the fluorescence signal generated as a function of time for various concentrations of miRNA-21. All experiments were performed in triplicate (n = 3) for each concentration. (C) Increased initial velocity v as a higher concentration of miRNA-21. The dashed line indicates the noise level (control average + 3σ) computed from the negative control.

To estimate initial velocity v, the linear phase from each concentration's time course was identified first, and then, the corresponding slope was computed by linear regression analysis. As the initial concentration of the miRNA-21 increased, the corresponding initial velocity v increased approximately linearly when using the decimal logarithmic scale for the concentration, as shown in **Figure S4C**. This result seemed to suggest that the collateral cleavage of the reporter via IMACC in the case of short RNAs follows the first-order kinetics of the Michaelis-Menten equation. The initial velocity v gradually increased as the concentration increased from $c = 10^2$ to 10^5 pM. The lowest concentration at $c = 10$ pM was lower than the noise level (red dashed line: control average + 3σ), suggesting CRISPR reaction at an early stage during ICP was not rapid at the lowest concentration. However, its intensity continuously increased over time and eventually became higher than the control, as already shown in Figure 3 and Figure S4B.

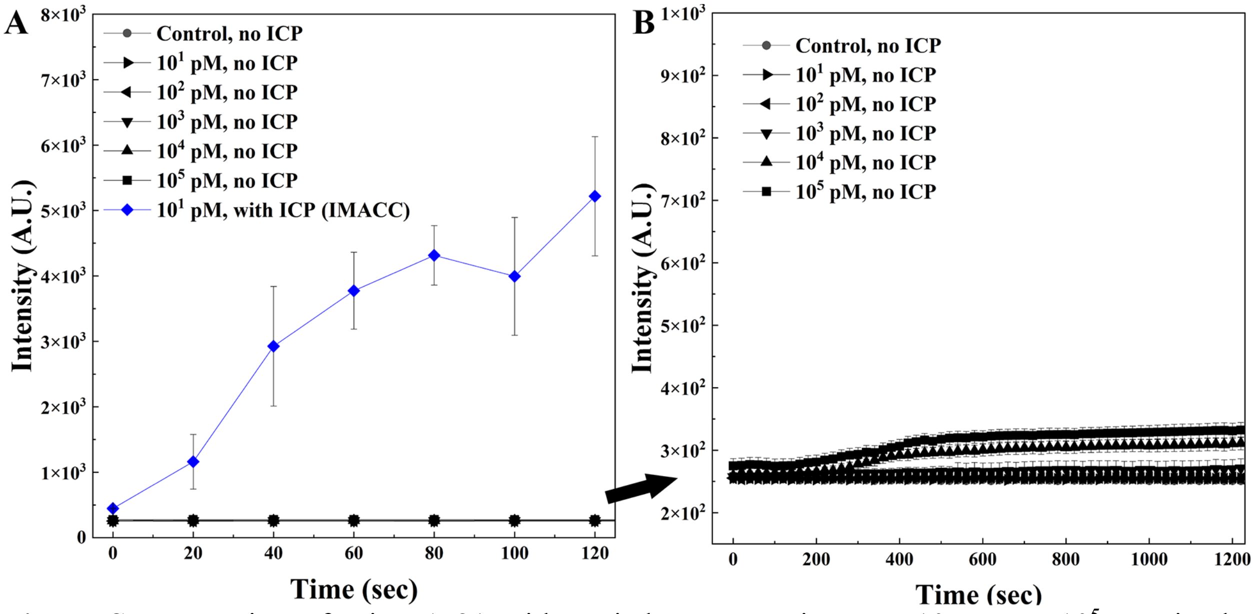

Figure S5. Detection of miRNA-21 with varied concentration c = 10 pM to 10^5 pM in the microfluidic channel based on diffusion only without ICP as a control experiment. The temperature was kept at 37 ℃ during the entire observation. (A) The fluorescence signals generated from inside the microchannel by varying the concentration of miRNA c = 10 pM to 10^5 pM without ICP (black symbols), and with ICP at 10 pM (blue, diamond). (B) The enlarged graphs from (A) only showing the fluorescence signals generated from the inside of the microchannel without ICP. Starting from about 500 seconds, only two of the highest concentrations, c = 10^4 pM and 10^5 pM showed relatively distinguishable signals as compared to the control. All the data were obtained from triplicate (n=3).

For comparison, we conducted the diffusion-based detection experiment in the microchannel and in a 384-well plate using the microplate reader. As demonstrated in **Figure S5** and **S6**, the diffusion-based assays in the microchannel required a relatively long-time $t \geq 500$ s to observe enough fluorescence signals from the two of the highest concentrations (10^4, 10^5 pM), and the rest of the lower concentrations still showed insufficient signal, leading to relatively lower sensitivity than IMACC within the given observation time. From the microwell plate, all the concentrations were detectable within observation time t = 9000 s, but it required more time to start to observe sufficient signals from low concentrations. In particular, at c = 10 pM, the signal became sufficiently observable after $t \geq 4000$ s compared to the control, demonstrating that the diffusion-based assay's detection speed is much slower than IMACC.

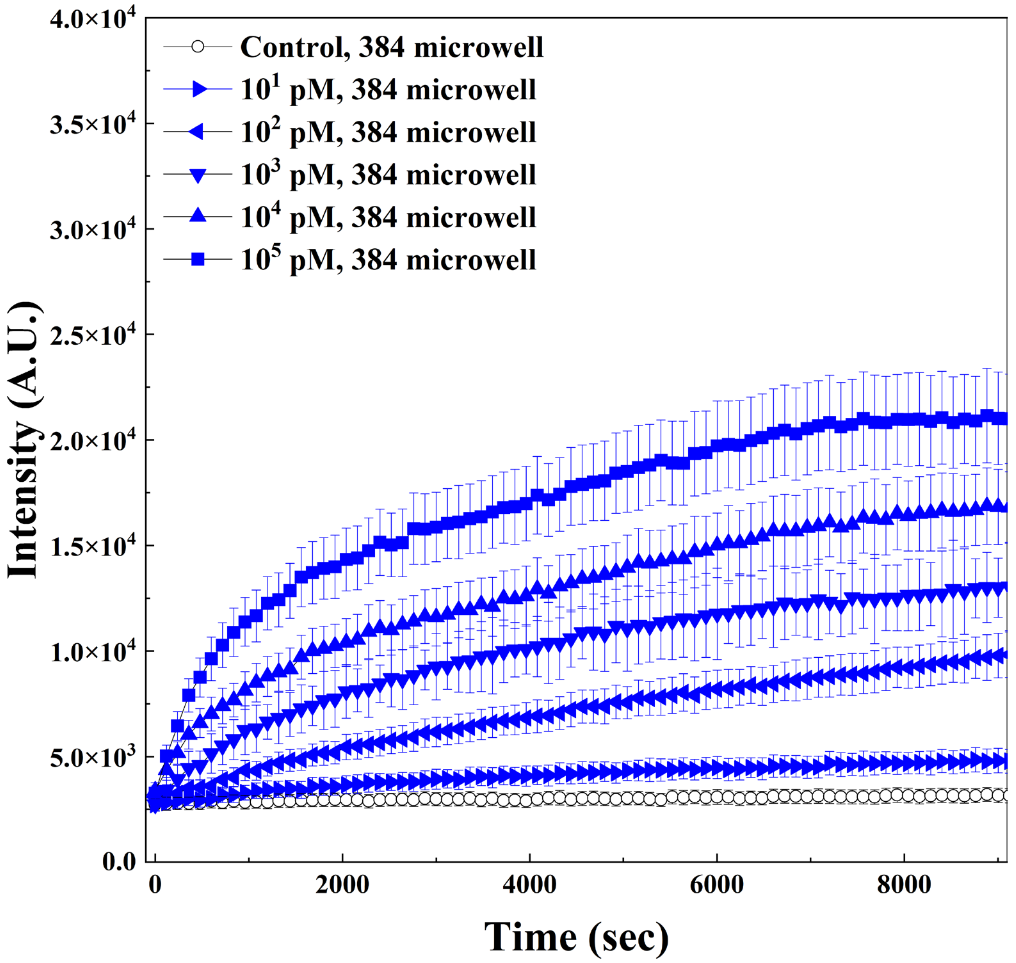

Figure S6. Detection of miRNA-21 with varied concentration c = 10 pM to 10^5 pM in a 384-microwell by using a microplate reader as a conventional approach. All the data were obtained from triplicate (n=3).

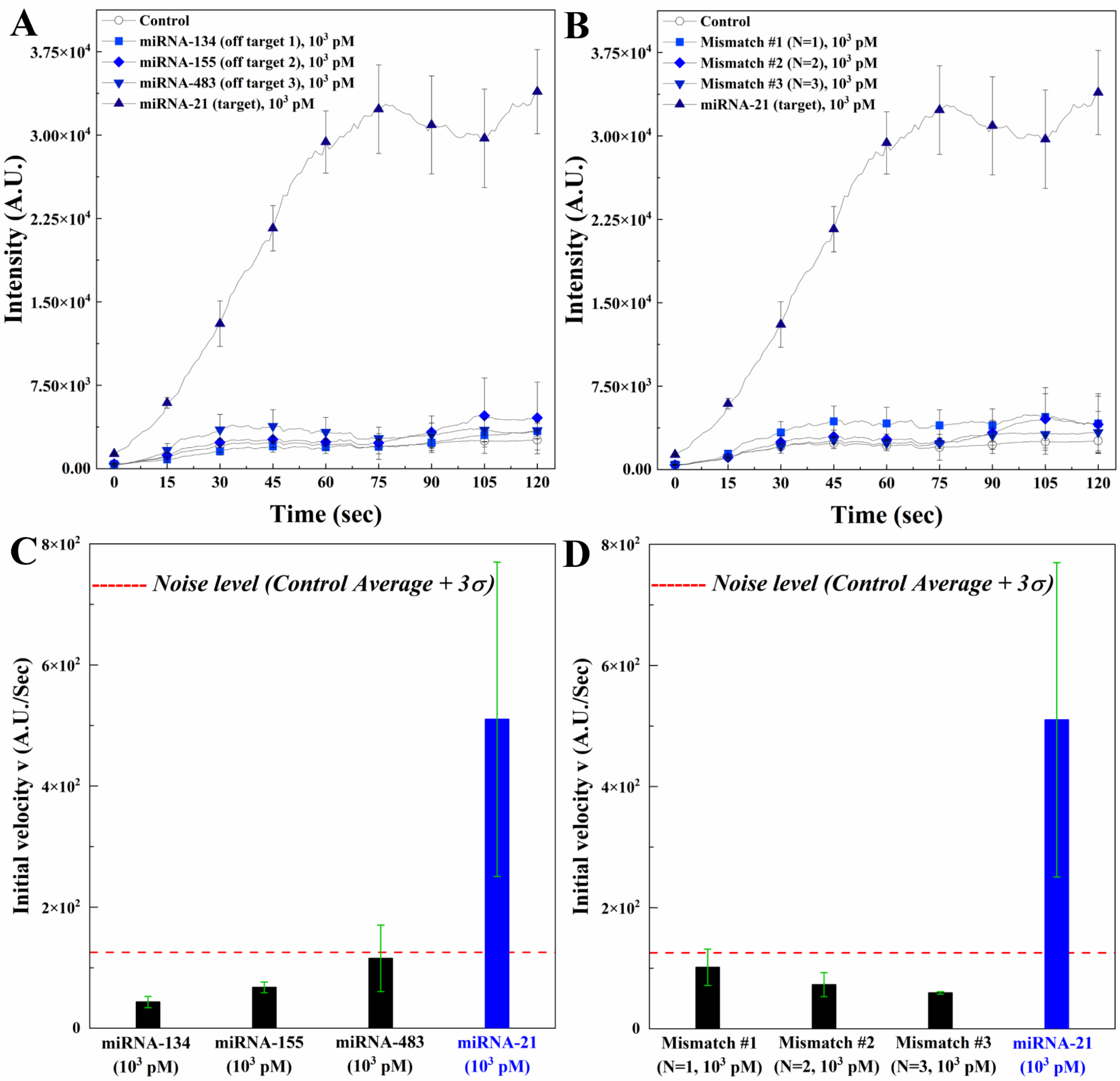

Figure S7. Investigation specificity of IMACC using off-targets or mismatch sequences. (A) The signals from all the off-targets (miRNA-134, 155, and 483) were much less than that of the target (miRNA-21). (B) The signal from different numbers of mismatches (N = 1,2, and 3) also was much less than the target (C) The initial velocity v obtained from all the off-targets (miRNA-134, 155, and 483: black bar) was much less than that of the target (miRNA-21: blue bar). (D) The initial velocity v obtained from different numbers of mismatches (N = 1,2, and 3: black bar) was also much less than the target (blue bar), demonstrating specific detection of IMACC.

Next, we investigate specificity of IMACC when there are off targets or mismatch sequences. For this, three different off-targets (miRNA-134, 155, and 483) and three different mismatch sequences (N = 1, 2, and 3) were prepared, and a comparison study with the complementary target, miRNA-

21, was conducted. The concentrations of all miRNAs were kept the same as $c = 10^3$ pM. First, the comparison study between three off-targets (miRNA-134, 155, and 483) and the target (miRNA-21) showed the signals generated over time during the ICP from all three off-targets were much less than that of targets, shown in **Figure S7A**. Similarly, the signal generated from mismatches was also less than that of the target, shown in **Figure S7B**. Although the signal decrease resulting from off-targets or mismatches did not show a clear dependency on the number of mismatches in the sequences, the target remained distinctly detectable against all other miRNAs, including off-targets and mismatched sequences. The initial velocity *v* computed from the intensities of both off-target and mismatch sequences over time also showed much lower, almost comparable to that of the noise level (control average + 3σ), shown in **Figures S7C** and **S7D**. These results indicate that IMACC specifically detected the intended target without affecting the specificity of CRISPR reaction, which is known as being sensitive to detecting a single mismatch (N = 1).

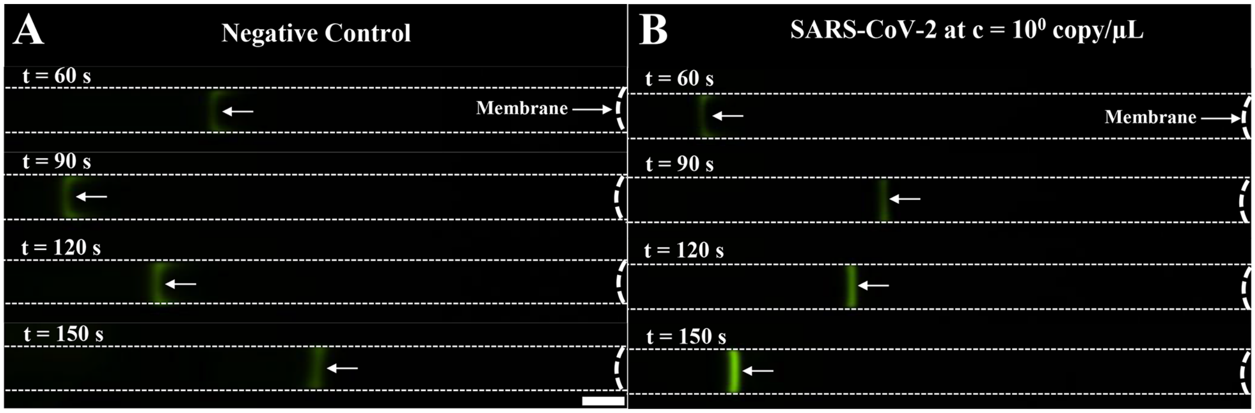

Figure S8. Time-lapse image showing fluorescence signal change of plugs generated from negative control and SARS-CoV-2 at c = 10^0 copy/μL in IMACC. (A) A slow increase of fluorescence signal from the negative control (B) Similarly, the signal increase of SARS-CoV-2 at c = 1 copy/μL was slow at an early phase of ICP. Then, a significant fluorescence signal increase was observed starting from 120 s, suggesting continuous accumulation of cleaved fluorescence reporter upon recognition and cleavage of target and thereby robust trans cleavage of the reporter. The white arrow indicates the location of the plug at each time point. The scale bar is 200 μm.

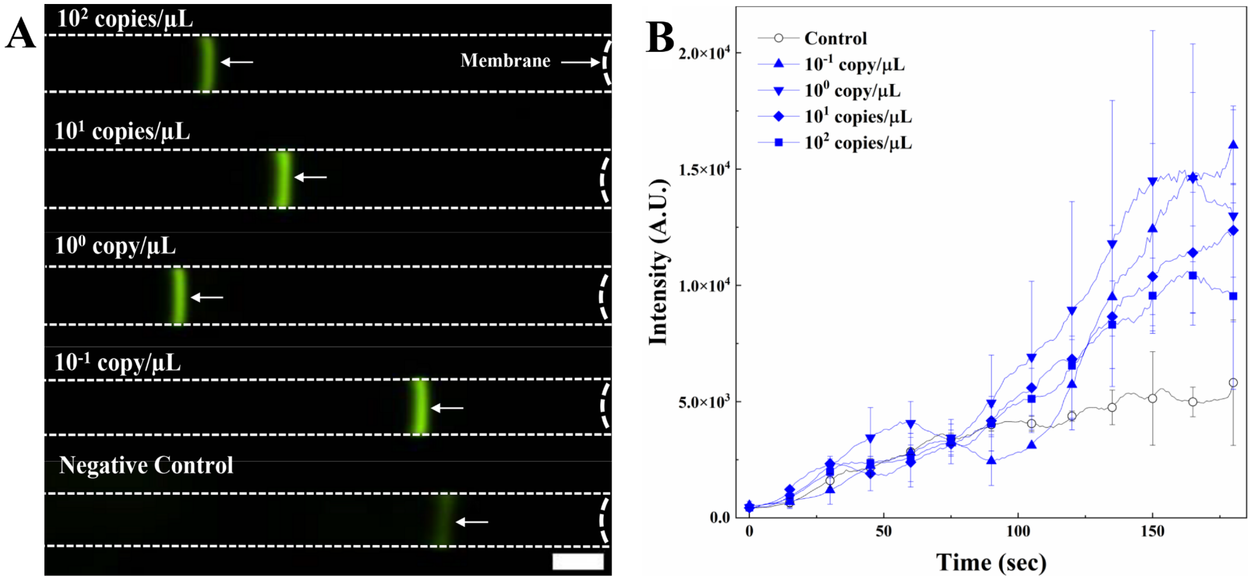

Figure S9. Observation of signal and quantitative analysis for detection of SARS-CoV-2 RNA by IMACC at the four representative copy numbers starting from c = 10^{-1} to 10^{2} copies/μL (A) Fluorescence images showing signal of plugs at t = 150 s from the four representative copy numbers of synthetic SARS-CoV-2 and negative control. The scale bar is 200 μm. (B) A graph showing the quantitatively analyzed fluorescence signal generated as a function of time during the detection of synthetic SARS-CoV-2 and negative control. All experiments were performed in triplicate (n = 3) for each concentration

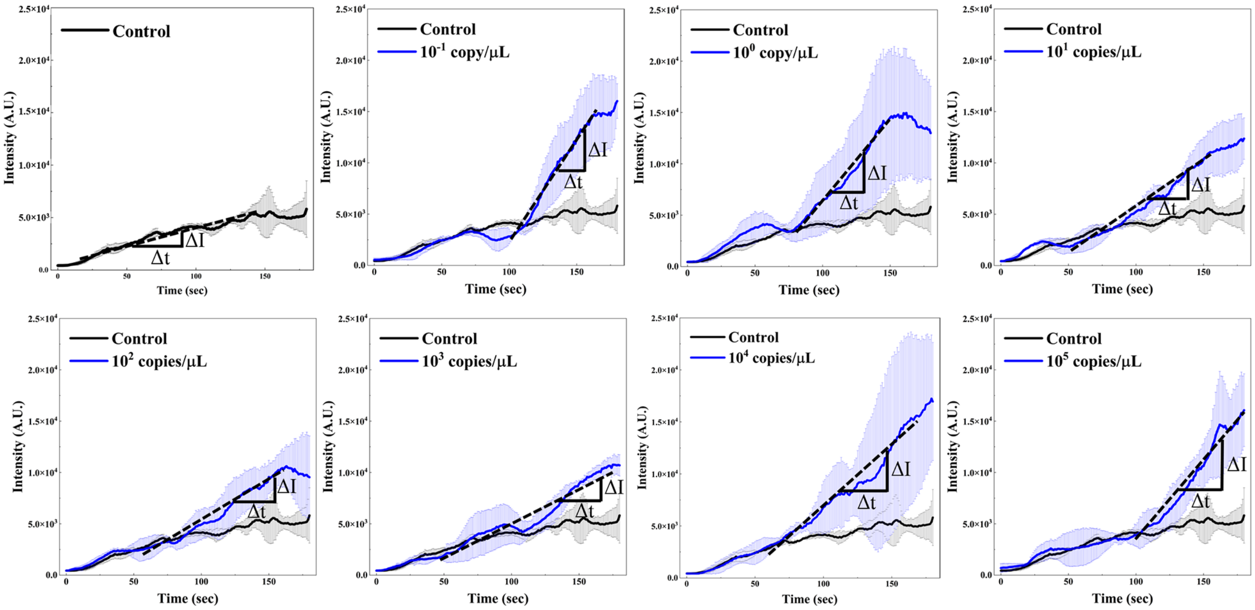

Figure S10. Detection of synthetic SARS-CoV-2 RNA at various concentrations $c = 10^{-1}$ to 10^{5} copies/μL using IMACC. The fluorescence intensity was measured as a function of time, demonstrating successful and reproducible detection of the SARS-CoV-2 RNA. All experiments were performed in triplicate ($n = 3$) for each concentration. In each graph, the dashed line represents the range used for linear regression analysis to estimate initial velocity v.

Figure S10 shows the detection of SARS-CoV-2 RNA at various concentrations $c = 10^{-1}$ to 10^{5} copies/μL using IMACC. As expected, strong fluorescence signals were observed from all 7 synthetic samples of the SARS-CoV-2 RNA than the negative control sample. The time points showing higher intensity for each positive sample were approximately $t = 110$ s for c = 10^{-1} copy/μL, and the time points for the rest of the other positive samples were earlier, between approximately $t = 70$ s – 110 s. Although there was a weak background signal generated from the negative control, the intensity was much less than that of the positive samples.

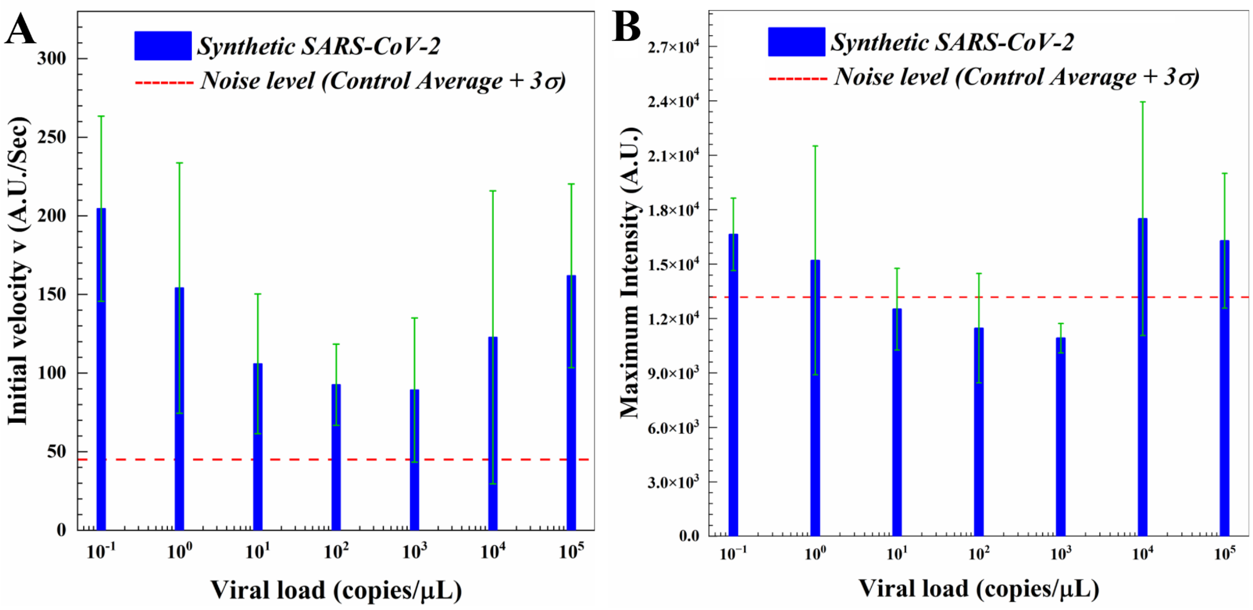

Figure S11. Quantitatively analyzed initial velocity *v* and maximum intensity computed from intensities of synthetic SARS-CoV-2. (A) The initial velocity *v* of the signal from the SARS-CoV-2 RNA was analyzed using linear regression, then compared to the noise level (control average + 3σ). (B) The maximum intensities compared to the noise level. Quantitative analysis confirmed large deviations for both initial velocities and maximum intensities, most of them overlapping with the noise level. Thus, statistically, the reliable detection of synthetic SARS-CoV-2 samples remained challenging at the current stage.

To further quantify the result, initial velocity *v* was calculated by linear regression after the retardation phase, as indicated by the dashed line in each graph, and maximum intensity was obtained from Figure S11. Unlike the miRNA-21, all positive samples of SARS-CoV-2 showed a retardation phase showing a slow increase of fluorescence signal, followed by a significant increase after a certain time of ICP. The significant intensity increase was observed at different time points throughout all positive samples, so the linear phase of each graph was determined individually according to each time point. Most interestingly, the initial velocity *v* and the maximum intensity showed a decreasing trend with increasing copy number and then increased again starting from $c = 10^4$ copies/μL.

In addition, most of the values for both the initial velocities v and the maximum intensities were not showing significance than noise level (control average + 3σ) and exhibited large

deviations. Statistically, the maximum intensities analysis confirmed that only 10^{-1} copy/μL of synthetic sample exceeded the noise level, but still their deviations were large. Therefore, reliable detection of synthetic SARS-CoV-2 samples remained statistically challenging at the current stage.

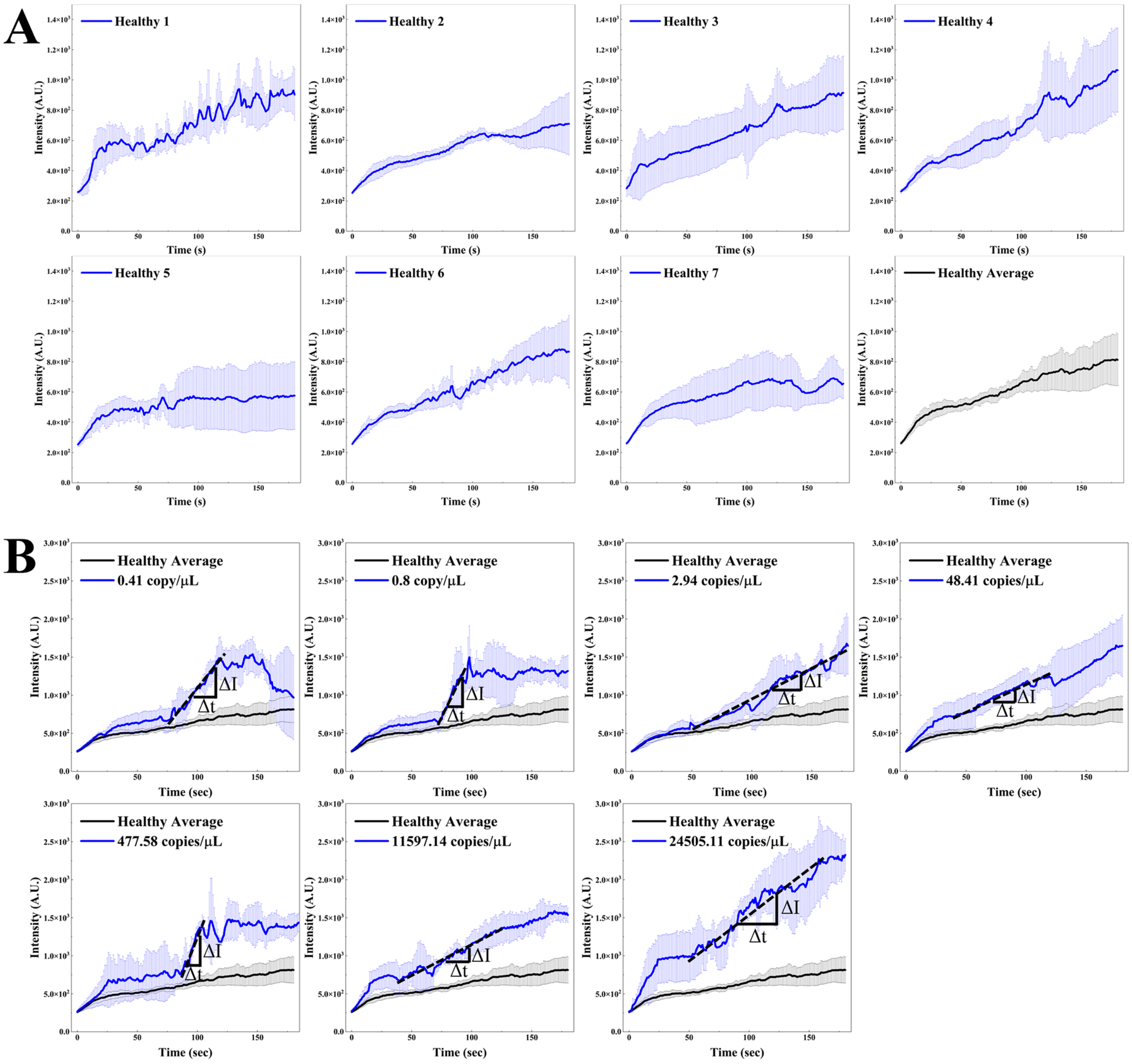

Figure S12. Detection of SARS-CoV-2 using IMACC from clinical samples. (A) Time-dependent fluorescence signals from 7 healthy samples (from healthy 1 to 7) and their average. (B) Time-dependent fluorescence signals from 7 SARS-CoV-2 infected patient samples (blue: 0.41, 0.8, 2.94, 48.41, 477.58, 11597.14, 24505.11 copies/μL) and healthy average (black). All data were obtained from triplicate tests.

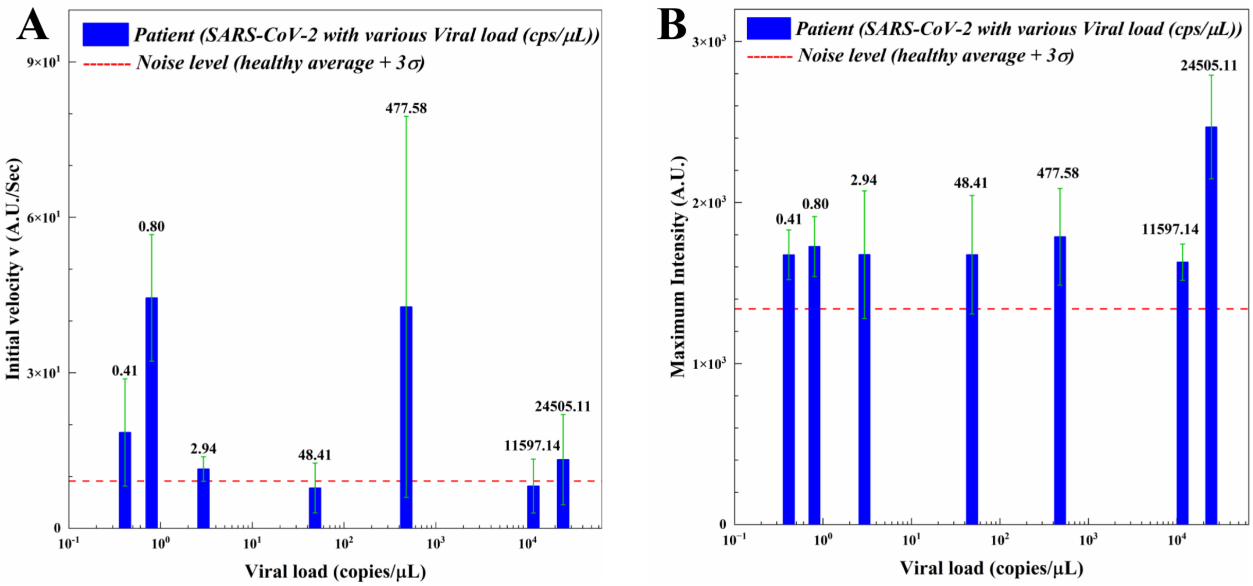

Figure S13. Quantitatively analyzed initial velocity ν and maximum intensity computed from intensities of SARS-CoV-2 in clinical samples. (A) The initial velocity *v* of 7 patient samples compared to the noise level (healthy average + 3σ). (B) The maximum intensities of 7 patient samples to the noise level. Similar to the synthetic samples, quantitative analysis indicated the values from both the initial velocities *v* and maximum intensities were not significantly high compared to the noise level due to the large deviations. Thus, statistically, the reliable detection of SARS-CoV-2 infected patient samples remained challenging at the current stage.

5. Supporting Movies

Movie S1. The movie shows the detection of miRNA-21 at c = 10 pM in IMACC. The signal intensity of the plug increases, and the width of the plug increases gradually as a function of time, demonstrating robust detection of short-length RNAs, the miRNA-21, in less than 2 minutes without enzymatic amplification. The movie is 10 FPS.

Movie S2. The movie shows the detection of SARS-CoV-2 at $c = 10^0$ copy/uL in IMACC. The signal intensity increases slowly as a function of time in the early phase. Then the signal significantly increases after approximately t = 110 s, allowing the detection of long-length RNAs, the SARS-CoV-2, within 2.5 minutes in an amplification-free manner. The movie is 10 FPS.

Movie S3. The movie shows the detection of SARS-CoV-2 at $c = 10^{-1}$ copy/uL in IMACC. The fluorescence signal increases slowly as a function of time in the early phase. Then the signal significantly increases after approximately t = 120 s, allowing the detection of long-length RNAs, the SARS-CoV-2, within 2.5 minutes in an amplification-free manner. The fluorescence signal near the end of the movie becomes relatively stronger than that of the signal from $c = 10^0$ copy/uL (Movie S2). This result fits well with the results demonstrated in Figure 4B, which shows the highest maximum intensity at the lowest copy number of the four positive samples. The movie is 10 FPS.

6. References

(1) LEE, Bom-yee; LEE, Bong-Kee. Surfactant-added hydrophilic polydimethylsiloxane (PDMS) as mold material for thermoplastic hot embossing process. AIP Advances, 2020, 10.9.

(2) WU, Zhigang; HJORT, Klas. Surface modification of PDMS by gradient-induced migration of embedded Pluronic. Lab on a Chip, 2009, 9.11: 1500-1503.

(3) Pham, V. S.; Li, Z. R.; Lim, K. M.; White, J. K.; Han, J. Y. Direct numerical simulation of electroconvective instability and hysteretic current-voltage response of a permselective membrane. *Phys Rev E* **2012**, *86* (4). DOI: ARTN 04631010.1103/PhysRevE.86.046310. Dang, V. T.; Pham, V. A numerical study of sample preconcentration using ion concentration polarization in single microfluidic channels with dual ion-selective membranes. *Aip Adv* **2023**, *13* (9), 095102. DOI: Artn 09510210.1063/5.0161190.

(4) Yeh, I. C.; Hummer, G. Diffusion and electrophoretic mobility of single-stranded RNA from molecular dynamics simulations. *Biophys. J.* **2004**, *86* (2), 681-689. DOI: Doi 10.1016/S0006-3495(04)74147-8.

(5) Zangle, T. A.; Mani, A.; Santiago, J. G. Theory and experiments of concentration polarization and ion focusing at microchannel and nanochannel interfaces. *Chem. Soc. Rev.* **2010**, *39* (3), 1014-1035. DOI: 10.1039/b902074h. Wei, X.; Panindre, P.; Zhang, Q.; Song, Y. A. Increasing the Detection Sensitivity for DNA-Morpholino Hybridization in Sub-Nanomolar Regime by Enhancing the Surface Ion Conductance of PEDOT:PSS Membrane in a Microchannel. *Acs Sensors* **2016**, *1* (7), 862-865. DOI: 10.1021/acssensors.6b00169.

(6) Kim, S. J.; Song, Y. A.; Han, J. Nanofluidic concentration devices for biomolecules utilizing ion concentration polarization: theory, fabrication, and applications. *Chem. Soc. Rev.* **2010**, *39* (3), 912-922. DOI: 10.1039/b822556g.
